# The Bio-herbicidal potential of some wild plants with allelopathic effects from Tabuk Region on selected local weed species

**DOI:** 10.3389/fpls.2023.1286105

**Published:** 2023-12-05

**Authors:** Amjad R. Alanaz, Eman A. S. Alatawi, Rahaf S. Alotaibi, Eman A. H. Alatawi, Attaf D. Albalawi, Hadeel A. Alhumayri, Qasem S. Alatawi, Basmah M. Alharbi

**Affiliations:** ^1^ Department of Biology, Faculty of Science, University of Tabuk, Tabuk, Saudi Arabia; ^2^ Genomic and Biotechnology Unit, Faculty of Science, University of Tabuk, Tabuk, Saudi Arabia

**Keywords:** allelopathy, weed control, phytochemical screening, weed species, allelochemicals, natural herbicides

## Abstract

Weeds are considered one of the most serious problems limiting global agricultural production. As a result, chemical herbicides have been extensively used for weed control. However, overuse of synthetic herbicides, has resulted in public concerns over the effect of herbicides on the health of the ecosystems and humans. In the food system, innovative approaches are needed to foster sustainable practices that preserve biodiversity, conserve habitats, and mitigate climate change factors. Thus, alternatives are required to control the weeds. This study aimed to determine the impact of some wild plants’ (*Citrullus colocynthis*, *Euphorbia retusa, Retama raetam, Artemisia monosperma, Tamarix gallica*, and *Artemisia judaica*) allelopathic potentials (at rates of 0, 15, 25, 35, and 50 g/L) on seed germination of selected local weeds (*Portulaca oleracea*, *Amaranthus retroflexus*, and *Chloris barbata*) collected from different locations in Tabuk region, Saudi Arabia. GC-MS analysis was used to evaluated the main compounds in the wild plants under study. The experimental design was completely randomized block design (CRBD) with three replicates. According to the results, phytochemical screening of the wild plants using GC-MS analysis showed a wide range of phytochemicals. *Amaranthus retroflexus* exhibited the highest germination rate in the control group. In addition, applying 5 g/L and 20 g/L of *Citrullus colocynthis* extracts had no discernible effect on the rate of germination of *A. retroflexus* seed; however, they were able to reduce the germination rate as compared to the control. As the extract concentration of *Artemisia monosperma* rose to 20 g/L, the germination rate of *A. retroflexus* dropped. Neither 35 g L-1 nor 50 g L-1 of *A. monosperma* extract inhibited the germinate of *A. retroflexus*. The germination rate of *Portulaca oleracea* decreased with increasing extract concentration of *C. colocynthis*. The extract of *C. colocynthis* at 50 g/L had the lowest rate. The germination rate of *Chloris barbata* decreased with increasing extract concentration of *C. colocynthis*. The highest germination rate of *C. barbata* was observed in control, followed by 5g/L, while no germination was observed at 20, 35, and 50 g/L of *C. colocynthis* extracts. *A. retroflexus’s* root length shrank when the extract concentration of *C. colocynthis* rose. *A. retroflexus’s* control sample had the most extended root length, followed by 5 and 20 g/L, respectively. *C. colocynthis* at 35 and 50 g/L showed no root elongation as this treatment inhibited radicle protrusion. High concentration of d-Glycero-d-galacto-heptose and pentane in *C. colocynthis* aqueous extract may be the cause of *C. colocynthis’* ability to inhibit *Chloris barbata* germination. The entire *C. barbata* plant length decreased when treated with 5 g/L of *C. colocynthis* extract. No growth was seen at any of the higher *C. colocynthis* concentrations (20, 35, and 50 g/L). The present work revealed that cultivating allelopathic crops like the selected studied wild plants from the Tabuk region has a promising future as an antagonistic species in a biological weed control program or combined with integrated weed management in agricultural food production.

## 1 Introduction

Weeds are considered one of the plant parasites that interfere with economic crop growth and development and cause eventually significant yield loss (Abdallah et al., 2021; Ansari et al., 2022; Khamare et al., 2022). Weeds compete with crops on light, nutrients, water, and space that diminishes crop growth and yield. Furthermore, weeds also harbor insect pests, bacterial, fungal, and viral pathogens, further decreasing the plant yield. With global population expected to reach over 9 billion by 2050, world food production cannot suffer from any reduction of yield by weed competition (Al-Harbi, 2017; Chauhan, 2020). Over-application of synthetic herbicides, however, has resulted in public concerns over the effect of synthetic herbicides on human health and the environment. Due to several environmental problems (such as the contamination of water and soil), health problems including chemical pollutants increasing the risk of human disease such as cancer (Walia et al., 2017) related to chemical herbicides, and insect resistance to pesticides, and resistance to parasitoids (Cimmino et al., 2015); many investigators have been suggesting alternative environmentally friendly methods for controlling the harmful weed. Applied plant extracts as eco-friendly compounds for controlling weeds are one of the promising method due to several bioactive compounds on those plants (Bohinc et al., 2020; Lengai et al., 2020).

Allelopathy is a phenomenon that has recently gained notice and is thought to be used in practice for weed and pest management. Many plants species experience allelopathic effects, which can be seen at any level of biological organization. In this phenomenon, a single creature generates certain types of specialized biochemical that have an impact on the natural vegetation of nearby plants and other species, including their growth and development, diversity, dominance, succession, and climax (Macías et al., 2014; El-Shora et al., 2022). Allelochemical-rich plant extracts can be used to control weeds and are a viable alternative to synthetic herbicides. However, allelopathy may affect the available resources in the environment. It occurs when one plant releases chemicals that affect the growth or survival of another plant species. This can lead to reduced competition for resources such as water, nutrients, and sunlight, allowing the allelopathic plant to dominate the area. However, it can also lead to a reduction in biodiversity and the overall health of the ecosystem (Başaran, 2021; Elkordy et al., 2022).

Allelochemicals are believed to be a joint action of several secondary metabolites including phenolic compounds, flavonoids, juglone and terpenoids. The inhibitory substances implicated in allelopathy are terpenoids and phenolic compounds (Abdeldaym et al., 2012; Al-Qahtan. 2019; Cocozza et al., 2021; Mahmoud et al., 2023). In the context of sustainable agriculture, the use of allelopathic and therapeutic plants has recently been proposed as a potential alternative pest and weed management method (Abdeldaym et al., 2014; Nasrine et al., 2014; Hemada and El-darier, 2015; Hozayn et al., 2015). Regarding the distinction between allelopathy and competition in particular, methodological issues hinder the research of allelopathy, which is still a contentious subject (Couso and Fernández, 2012; Cocozza et al., 2021 Al-Harbi, 2021). There is a lack of knowledge on the significance of allelochemicals in plant survival in arid and semiarid climates when combined with allelopathy. In Saudi Arabia’s dry regions, six wild species (*Citrullus colocynthis, Retama raetam, Euphorbia retusa, Artemisia monosperma, Tamarix gallica* and *Artemisia judaica*) were evaluated for controlling three weed species namely, *Portulaca oleracea, Amaranthus retroflexus*, and *Chloris barbata*. In this study, our hypothesis is that our wild plants could have some compounds to reduce the growth of some local weeds. Therefore, the current study is aimed to determine the allelopathic potential of some wild plants in the Tabuk region, Saudi Arabia and assess their ability to suppress local weed growth.

## 2 Materials and methods

### 2.1 Collection of studied plant materials

**Figure 1:**
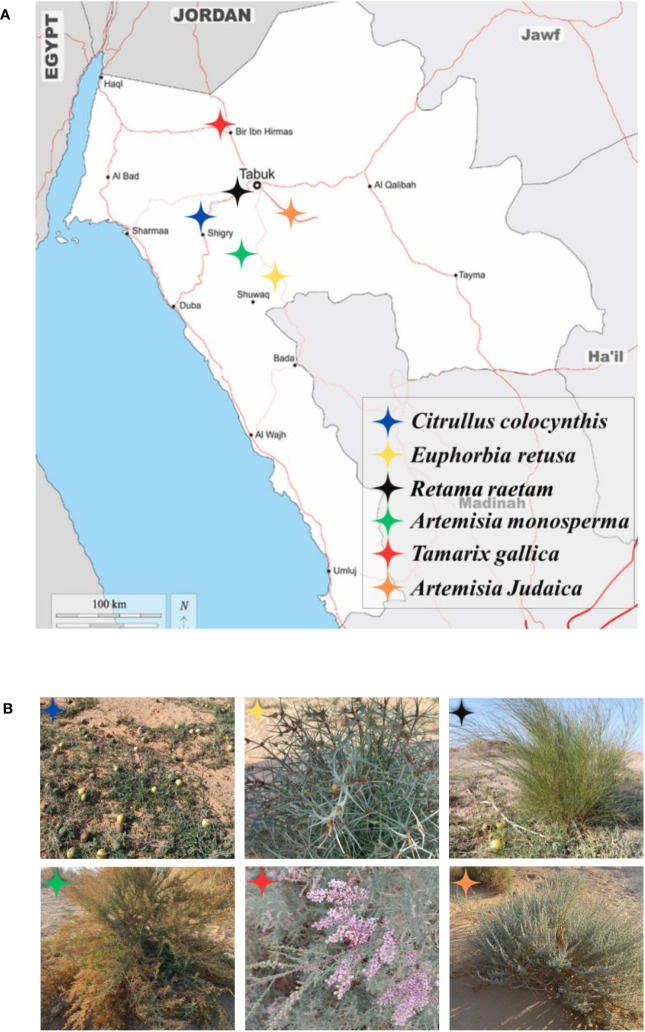
**(A)** The map of the Tabuk Region showing locations of studied donor wild plants. **(B)** The phenotype of studied donor wild plants in their natural habitats during the period from December 2022 to January 2023, starting from the top row on the left; *Citrullus colocynthis, Euphorbia retusa, Retama raetam, Artemisia monosperma, Tamarix gallica and Artemisia judaica* respectively.

Fresh and healthy aerial parts of wild plants chosen for the current study (*Citrullus colocynthis*, *Retama raetam*, *Euphorbia retusa*, *Artemisia monosperma*, *Tamarix gallica* and *Artemisia judaica*) were collected during the vegetative and flowering stages (December 2022 – January 2023) from different habitats in Tabuk **Figure 1**. Seeds of selected weedy species used for the investigation (*Portulaca oleracea, Amaranthus retroflexus, and Chloris barbata*) were collected from different local locations in Tabuk (farms and roadsides or purchased from Tabuk local market). All specimens (donor plants and receptor plants) were authenticated by a renowned taxonomist.

### 2.2 Preparation of plant materials

Collected aerial parts of plant materials of the six studied wild plants were washed with running tap water, and then they were dried in an oven with air circulation at 35°C until a steady dry mass is reached. The dried aerial parts were then grounded into a fine powder separately using an electrical grounder. Powdered materials were stored in airtight containers to protect them from humidity and then kept at room temperature until use.

### 2.3 Preparation of aqueous extracts

To prepare stock aqueous extracts of 50 g L^-1^, 50 g of plant dry material was weighed and transferred to a clean flask, and 1000 ml of autoclaved distilled water was added to each of the flasks. Mixtures were then homogenized by gently shaking the flask periodically using an electrical shaker at room temperature 20 ±2°C for 48 hours, and then they were left to rest for 24 hours. After this period, the mixtures were filtered through thick cotton layers to remove plant cellular debris and other residues using the Buechner funnel supplied with a suction pump. Different dilutions (5 g L^-1^, 20 g L^-1^, and 35 g L^-1^) were prepared from the stock solution. All solutions were then kept at 5°C in the dark until further use.

### 2.4 Phytochemical screening

To prepare the studied plant material for GC-MS analysis, plant powder (100 g) was de-fatted using 75% methanol as the solvent. The solid–liquid mixture was filtered with Whatman filter paper number 2, and the residue was dried in an oven at 50°C for six hours. The dried defatted plant sample was stored at 4°C before extraction. Maceration was carried out for 24 hours using a solvent-aqueous mixture containing ethanol, methanol, acetone, and ethyl acetate, followed by sonication with heating for 1 hour. The extract was then filtered and finally injected. GC-MS for identification of phytocomponents was conducted using the database of the National Institute Standard and Technology MS library (NIST- MS library). Samples were extracted in Methano Maceration was carried out for 24 hrs followed by sonication with heating for 1 hour Extract was filtered and inected (Restek HP-5-MS 30-0.25 0.25 Agilent Technologies GC/MS System GC 7890B).

### 2.5 *In vitro* seed germination bioassay

The Petri-dish experiment was carried out to investigate the potential allelopathic effects of wild plant species’ aqueous extracts on germination percentage, seedling morphology, as well as plumule and radicle lengths of the weedy species. The seeds of weeds were sterilized with a 10% sodium hypochlorite solution for 5 minutes, then rinsed with distilled water several times, and kept in the refrigerator for 24 hours to ensure uniform germination. Additionally, 9-cm diameter Petri-dishes and disks of Whatman No. 1 filter papers were autoclaved at 120°C for 15 minutes.

In a Petri dish containing two filter paper discs, 20-30 seeds of the three studied weeds were distributed. Plates were moistened with 7 ml of each extract or distilled water for the control treatment. Subsequently, plates were placed on the bench top at room conditions with a day temperature of 22 ±2°C and a night temperature of 16 ±2°C. Three biological replicates were allocated per each treatment and different treatments were arranged in a complete randomized block design. Plates were monitored daily for ten days to detect seed germination. At the end of the experiment, after 10 days, the germination percentage was calculated using the following formula:


$$Germination\;percentage=\;\frac{Number\;of\;germinated\;seeds}{Total\;number\;of\;sown\;seeds}x\;100$$

(Aniat-Ul-Haq and Agnihotri, 2010).

The plumule and radicle growth was measured using ImageJ software, National Institutes of Health, USA.

### 2.6 Statistical analysis

SPSS software was used for all statistical work. The Shapiro-Wilk test was used to determine whether the data were normally distributed, and the parametric test, the T-test, was used on normally distributed data while the non-parametric test, the Mann-Whitney test, was used on non-normal data. In all statistical tests, the significance of differences was determined if the *P* value was ≤ 0.05 according to Tukey’s test.

The design of laboratory experiment was completely randomized. The normality of the distribution (Shapiro-Wilk test, p>0.05) and the homogeneity of variances (Cochran test, p>0.05) of the data were tested. A Two-way ANOVA followed by Tukey’s test (p>0.05) was used to determine significant differences between the extracts of wild plants and seed germination of local weed species. SPSS software was used for all statistical analysis.

## 3 Results and discussion

### 3.1 Phytochemical composition

**Figure 2:**
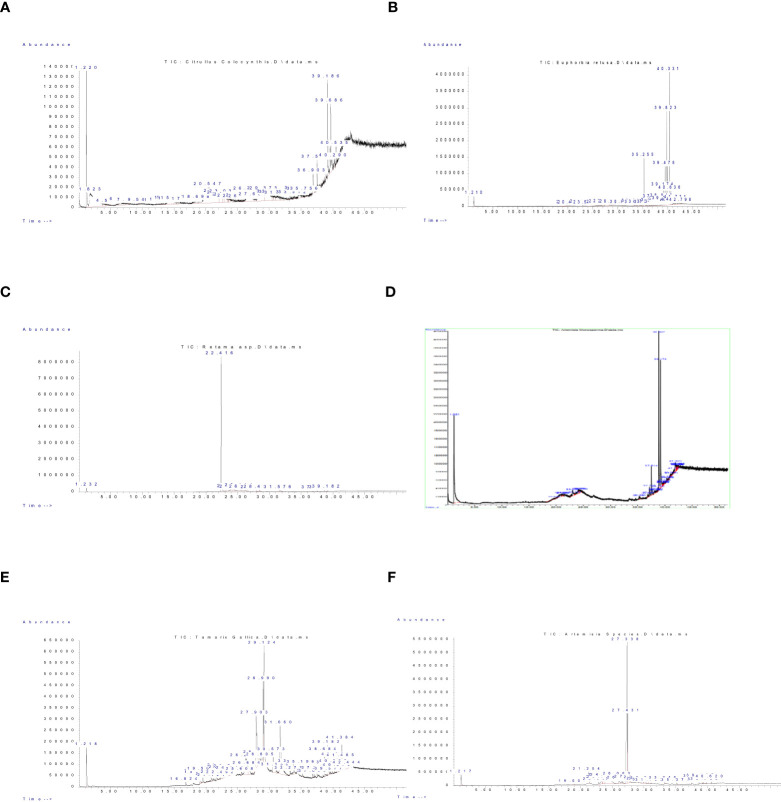
GC-MS chromatogram of Components detected in the extract of studied wild plants. **(A)** *Citrullus colocynthis*, **(B)** *Euphorbia retusa*, **(C)** *Retama raetam*, **(D)** *Artemisia monosperma*, **(E)** *Tamarix gallica*, and **(F)** *Artemisia judaica*.

Phytochemical analysis of aerial parts from wild plants studied in the current work; *Citrullus colocynthis*, *Retama raetam*, *Euphorbia retusa*, *Artemisia monosperma*, *Tamarix gallica* and *Artemisia judaica* are shown in **Figure 2** and **Supplementary Tables 1–6** respectively, along with more details on detected compounds in **Supplementary Tables 7–12** in the **Appendices**. Results indicate that these plants are rich in a wide range of important bioactive phytochemicals including phenols, steroids (stigasta-7,25-dien-3-ol,3.beta.,5.alpha., 9,19-Cyclolanostan-3-ol, 24-methylene-, (3.beta), and alkaloids (Salama, 2012; El-amier et al., 2021; Romeilah et al., 2021; Awada et al., 2022). Erida et al. (2023) reported that there is a positive correlation between the aforementioned allelochemical compounds and the seed germination of some herbaceous plants. Whereas these compounds are able to inhibit seed germination and weed growth by inhibiting auxin activity during cell division and elongation.

### 3.2 The effect of extracts on seed germination rate

#### 3.2.1 Response of *Amaranthus retroflexus* to treatment with wild plant extracts

**Figure 3:**
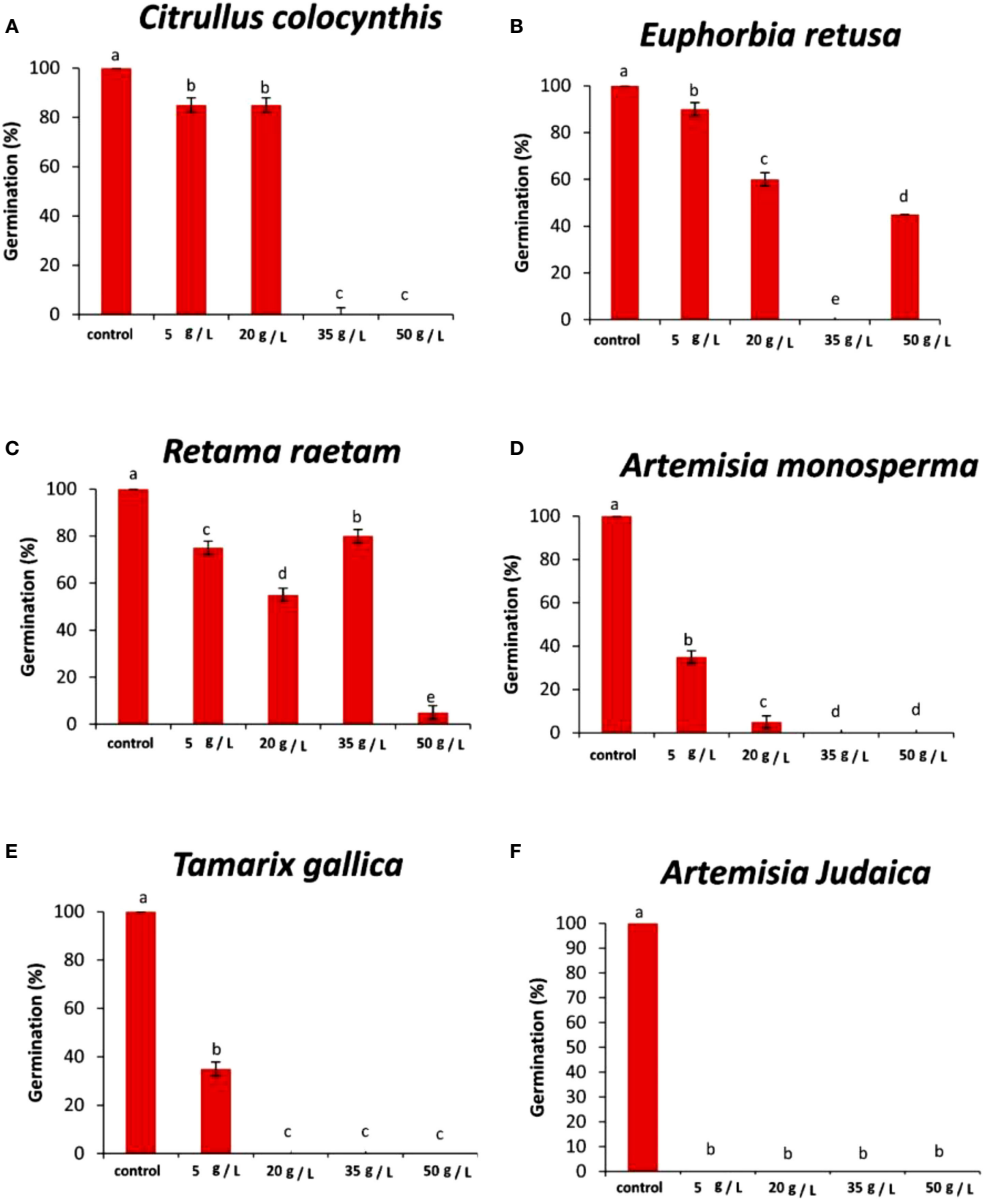
Effect of different extract concentrations of studied wild plants on the germination rate of *Amaranthus retroflexus.* **(A)** *Citrullus colocynthis*, **(B)** *Euphorbia retusa*, **(C)** *Retama raetam*, **(D)** *Artemisia monosperma*, **(E)** *Tamarix gallica* and **(F)** *Artemisia judaica* extracts. Values are the mean ± SE of two biological replicates each with 30 seeds. Bars of the graphs represent standard errors. Significant differences in means are indicated by different letters according to Tukey’s test (*P*≤0.005).

The data in **Figure 3A** indicated that the germination rate of *Amaranthus retroflexus* decreased with increasing extract concentration of *Citrullus colocynthis*. The highest germination rate of *Amaranthus retroflexus* was observed in control, while there was no significant difference observed in *Amaranthus retroflexus* seed germination rate as a result of applying 5 g/L and 20 g/L of a *Citrullus colocynthis* water extracts. Additionally, *Amaranthus retroflexus* seed germination was completely inhibited as a result of treatments with *Citrullus colocynthis* at 35 g/L and 50 g/L. These results agree with previous results reported on applying *Citrullus colocynthis* extracts at a concentration of 5%, as well as all other organ extracts at 10% concentration, which fully suppressed ryegrass seed germination. This results could be due to the compounds of *Citrullus colocynthis* such as glycosides, alkaloids, and *cucurbitacins* (Mseddi et al., 2018). The effect of *Citrullus colocynthis* extract on reducing the germination of *Amaranthus retroflexus* observed in the present work could be due to the high content of d-Glycero-d-galacto-heptose and Propane in *Citrullus colocynthis* extract (**Figure 2A**).

The data presented in **Figure 3B** suggests that the germination rate of *Amaranthus retroflexus* decreased as the extract concentration of *Euphorbia retusa* increased until 35 g/L where seed germination was completely inhibited and then increased at 50 g/L but this was represented by only radicle protrusion. The control of *Amaranthus retroflexus* seeds treated with distilled water exhibited the highest germination rate. However, no germination was observed in *Amaranthus retroflexus* seeds at 35 g/L of *Euphorbia retusa* extract. These results are in agreement with the previous study conducted by El-amier et al. (2021) that proved the significant phytotoxic effect of *Euphorbia retusa* against the germination and seedling growth of *Chenopodium murale* at different plant extract concentrations (2.5 to 40 mg ml^-1^) over control. The effect of *Euphorbia retusa* extract on reducing the germination of *Amaranthus retroflexus* herein could be due to the high content of 9,19-Cyclolanost-24-en-3-ol, (3.beta.) and 9,19-Cyclolanostan-3-ol, 24-methylene-, (3.beta.) in *Euphorbia retusa* water extracts (**Figure 2B**).

**Figure 3C** indicates that the germination rate of *Amaranthus retroflexus* decreased with increasing *Retama raetam* extract concentration until 20 g/L, after which it increased at 35 g/L and then decreased to the minimum at 50 g L^-1^ of *Retama raetam*. The highest germination rate of *Amaranthus retroflexus* was observed in the control. It has been found that *Retama raetam* crude powder reduced the seedling length, fresh weight, and dry weight of two seeds (*Triticum aestivum* and *Phalaris minor*) in pure and mixed cultures studies 21 days after sowing (El-darier et al., 2018). The effect of *Retama raetam* extract on reducing the germination of *Amaranthus retroflexus* might be due to the high content of Dodecahydro-7,14-methanodipyrido[1,2-a:1’,2’-e][1,5]diazocine in *Retama raetam* extract as GC-MS analysis results revealed (**Figure 2C**). According to the data presented in **Figure 3D**, the germination rate of *Amaranthus retroflexus* decreased as the extract concentration of *Artemisia monosperma* increased until 20 g/L. The control exhibited the highest germination rate and no germination of *Amaranthus retroflexus* was recorded at 35 g L^-1^ and 50 g L^-1^ of *Artemisia monosperma* extract. In this regard, Abd-ElGawad et al. (2023) reported that the extracted essential oils of the *Artemisia monosperma* samples showed significant inhibition in the seed germination of the weed *Dactyloctenium aegyptium* and the crop *Lactuca sativa*, however, the weed was more resistant to the allelochemicals. The effect of *Artemisia monosperma* extract detected in the present work on the germination of *Amaranthus retroflexus* might be due to the high content of 2-Methylamino-N-phenyl-acetamide, 17-Pentatriacontene, and gamma-Sitosterol diazocine in *Artemisia monosperma* extract (**Figure 2D**). Additionally, some studies have shown that *Artemisia monosperma* produces a variety of secondary metabolites that have allelopathic properties such as camphor, cineole, and alpha-pinene (Ivănescu et al., 2021).

**Figure 3E** reveals that the germination rate of *Amaranthus retroflexus* decreased with increasing concentration of *Tamarix gallica* extract up to 5 g/L as compared to the control seeds that had the highest germination rate. No *Amaranthus retroflexus* seed germination was observed when treated with 20 g/L of *Tamarix gallica* extract or higher. There have been no reports on the allelopathic effect of *Tamarix gallica* plant on weedy seeds, even though it has historically been used to treat a variety of diseases and is sold as a herbal medication in many nations due to its bioactive contents (Ksouri et al., 2009; Tabassum et al., 2013). The effect of *Tamarix gallica* extract on reducing the germination of *Amaranthus retroflexus* in the current work might be due to the high content of Androst-5-en-17-one, 3-(acetyloxy)-19-hydroxy-, (3.beta.), p-Dimethylaminobenzylidene p-anisidine, and Benzene, 1-(1-buten-3-yl)-2-vinyl- diazocine in *Tamarix gallica* extract (**Figure 2E**). Also, previous studies have shown that *Tamarix gallica* produces a variety of bioactive compounds, including phenolic acids, flavonoids, and terpenes (Khan et al., 2018). The data presented in **Figure 3F** suggests that there was no germination of *Amaranthus retroflexus* observed with all tested concentrations of *Artemisia judaica* compared to the control. In accordance with our results, Hashem et al. (2019) evaluated the effect of *Artemisia judaica* root exudates and aqueous leaf extracts at three different concentrations (25, 50, and 100 μg/ml) on two weed plants (*Portulaca oleracea* and *Phalaris minor*) to determine their allelopathic effects. They mentioned that in a lab trial, *Artemisia judaica* had herbicidal effects that considerably decreased the growth of both weeds’ seedlings, and their ability to germinate.

It has been found that *Artemisia judaica* produces allelopathic compounds that can affect the germination, growth, and survival of other plant species (Khalid et al., 2023). The effect of *Artemisia judaica* extract on reducing the germination of *Amaranthus retroflexus* might be due to the high content of Naphtho[1,2-b] furan-2,6(3H,4H)-dione, 3a,5,5a,9,9a,9b-hexahydro-9-hydroxy-3,5a,9-trimethyl in *Artemisia judaica* extract (**Figure 2A**). Moreover, it has been found that *Artemisia Judaica* produces a variety of secondary metabolites that have allelopathic properties such as camphor, terpinen-4-ol, and alpha-pinene (Yosef Friedjung et al., 2013).

#### 3.2.2 Response of *Portulaca oleracea* to treatment with wild plant extracts

**Figure 4:**
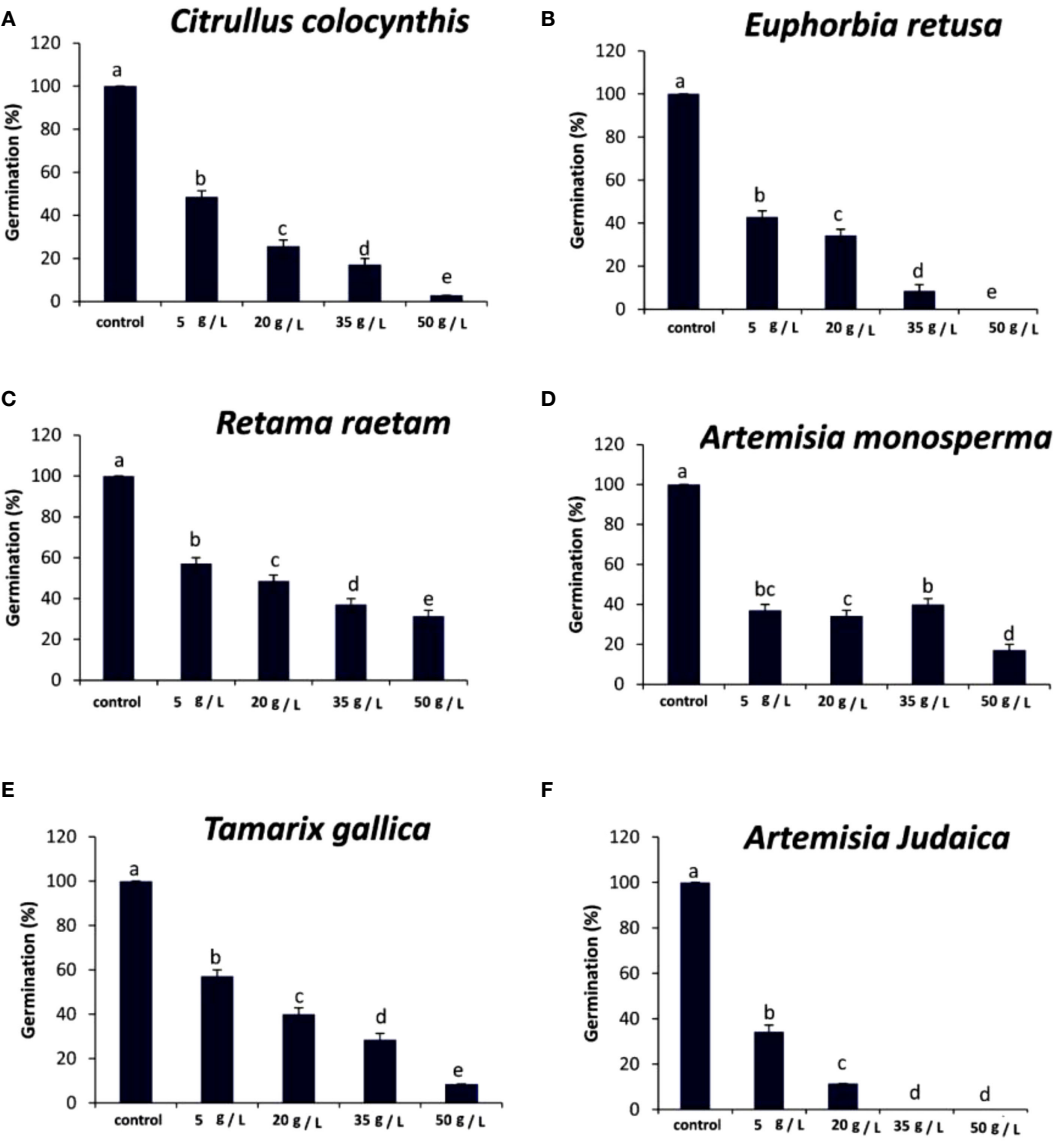
Effect of different extract concentrations of studied wild plants on the germination rate of *Portulaca oleracea.* **(A)** *Citrullus colocynthis*, **(B)** *Euphorbia retusa*, **(C)** *Retama raetam*, **(D)** *Artemisia monosperma*, **(E)** *Tamarix gallica* and **(F)** *Artemisia judaica* extracts. Values are the mean ± SE of two biological replicates each with 30 seeds. Bars of the graphs represent standard errors. Significant differences in means are indicated by different letters according to Tukey’s test (*P*≤0.005).

The data in **Figure 4A** indicated that the germination rate of *Portulaca oleracea* decreased with increasing extract concentration of *Citrullus colocynthis*. The highest germination rate of *P. oleracea* observed was in control, while the lowest germination rate was observed at 50 g/L of *C. colocynthis* extract. Previous studies indicated the allelopathic effects of *C. colocynthis* on the growth of the shoot and root of *Hordeum vulgare* (Salama and Al Rabiah, 2015). The effect of *C. colocynthis* extract on reducing the germination of *P. oleracea* could be due to the high content of d-Glycero-d-galacto-heptose and Propane in *C. colocynthis* plant aqueous extract (**Figure 2A**). The information presented in **Figure 4B** suggests that the germination rate of *Portulaca oleracea* decreased as the extract concentration of *Euphorbia retusa* increased. The control of *Portulaca oleracea* seed exhibited the highest germination rate. However, no germination was recorded in *Portulaca oleracea* seeds when 50 g/L of *Euphorbia retusa plant* extract was applied. In this regard, El-amier et al. (2021) indicated that *Euphorbia retusa* methanolic extract from aerial parts at different tested concentrations significantly inhibited the development of *Chenopodium murale* seedlings compared to the control. They added that *Chenopodium murale* seedling growth is more sensitive to the applied *Euphorbia retusa* extract than seed germination (El-amier et al., 2021). The effect of *Euphorbia retusa* extract on reducing the germination of *Portulaca oleracea* could be due to the high content of 9,19-Cyclolanost-24-en-3-ol, (3.beta.) and 9,19-Cyclolanostan-3-ol, 24-methylene-, (3.beta.) in *Euphorbia retusa* extract (**Figure 2B**).

**Figure 4C** indicates that the germination rate of *Portulaca oleracea* gradually decreased with increasing the extract concentration of *Retama raetam*. The highest germination rate of *Portulaca oleracea* was observed in the control. El-darier et al. (2018) found that crude powder of the aerial shoots of *Retama raetam* suppressed the germination of both *Triticum aestivum* and *Phalaris minor*. The effect of *Retama raetam* extract on reducing the germination of *Portulaca oleracea* might be due to the high content of Dodecahydro-7,14-methanodipyrido[1,2-a:1’,2’-e][1,5]diazocine in *Retama raetam* extract (**Figure 2C**). According to the information presented in **Figure 4D**, the germination rate of *Portulaca oleracea* decreased as the extract concentration of *Artemisia monosperma* increased. The control exhibited the highest germination rate, and no significant difference was observed between 5, 20, and 35 g/L while the lowest significant germination rate was observed at 50 g L^-1^. It has been reported that certain *Asteraceae* species have allelopathic effects on other plant species, decreasing seed germination and subsequent small grain crops’ emergence (Abu-Romman, 2011). Many isolated *Artemisia monosperma* components have been shown to have antimicrobial activity against 12 -lipoxygenase, colorectal and breast cancer cell lines, Mycobacterium, and *Staphylococcus aureus* (Hijazi and Salhab, 2010). The effect of *Artemisia monosperma* extract on reducing the germination of *Portulaca oleracea* might be due to the high content of 2-Methylamino-N-phenyl-acetamide, 17-Pentatriacontene, and gamma-Sitosterol diazocine in *Artemisia monosperma* extract (**Figure 2D**). Additionally, the most probable explanation for the reductions in germination is the presence of allelochemicals in the aqueous extracts (Javaid and Anjum, 2006). Also, Ivănescu et al. (2021) found that *Artemisia monosperma* produces a variety of secondary metabolites that have allelopathic properties such as camphor, cineole, and alpha-pinene. These previous compounds could work as fungicides and herbicides.

**Figure 4E** reveals that the germination rate of *Portulaca oleracea* gradually decreased with increasing extract concentration of *Tamarix gallica* extract. The control group had the highest germination rate, and 50 g/L of *Tamarix gallica* extract showed a lower germination rate. The effect of *Tamarix gallica* extract on reducing the germination of *Portulaca oleracea* might be due to the high content of Androst-5-en-17-one, 3-(acetyloxy)-19-hydroxy-, (3.beta.), p-Dimethylaminobenzylidene p-anisidine, and Benzene, 1-(1-buten-3-yl)-2-vinyl- diazocine in *Tamarix gallica* extract (**Figure 2E**). These compounds could be released into the soil and has allelopathic effects (Khalid et al., 2023). Previous studies have shown that *Tamarix gallica* produces a variety of allelopathic compounds, including phenolic acids, flavonoids, and terpenes (Khan et al., 2018).

The data presented in **Figure 4F** suggests that the germination rate of *Portulaca oleracea* decreased with increasing extract concentration of *Artemisia judaica* extract until 20 g/L while there was no germination of *Portulaca oleracea* observed at 35 and 50 g/L concentrations of *Artemisia judaica.* Our results are in agreement with previous study that have shown that *Artemisia Judaica* produces a variety of secondary metabolites that have allelopathic properties such as camphor, terpinen-4-ol, and alpha-pinene (Yosef Friedjung et al., 2013). The effect of *Artemisia judaica* extract on reducing the germination of *Portulaca oleracea* might be due to the high content of Naphtho[1,2-b] furan-2,6(3H,4H)-dione, 3a,5,5a,9,9a,9b-hexahydro-9-hydroxy-3,5a,9-trimethyl in *Artemisia judaica* extract (**Figure 2A**).

#### 3.2.3 Response of *Chloris barbata* to treatment with wild plant extracts

**Figure 5:**
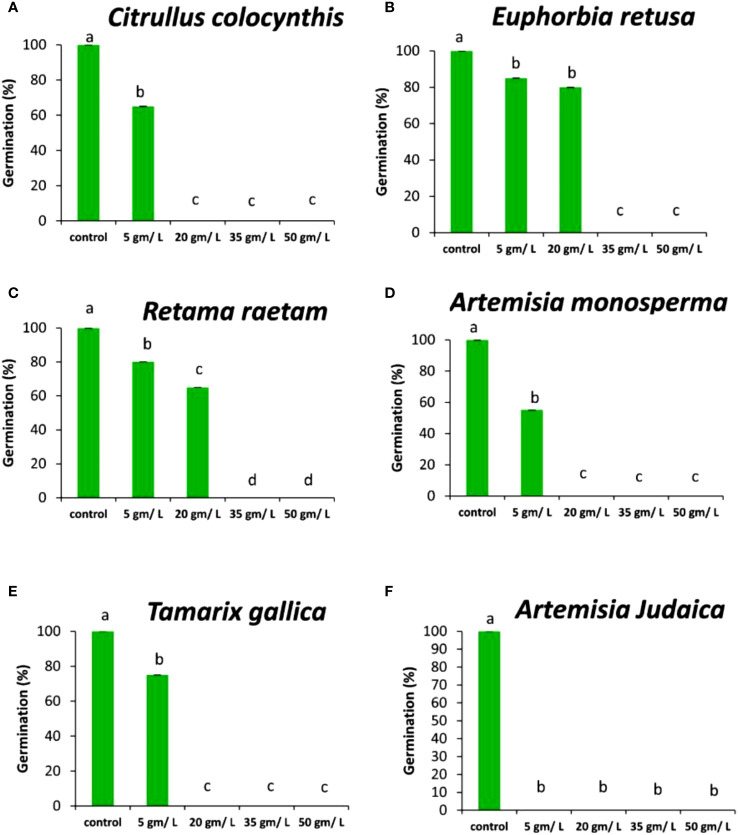
Effect of different extract concentrations of investigated wild plants on the germination rate of *Chloris barbata.* **(A)** *Citrullus colocynthis*, **(B)** *Euphorbia retusa*, **(C)** *Retama raetam*, **(D)** *Artemisia monosperma*, **(E)** *Tamarix gallica* and **(F)** *Artemisia judaica* extracts. Values are the mean ± SE of two biological replicates each with 30 seeds. Bars of the graphs represent standard errors. Significant differences in means are indicated by different letters according to Tukey’s test (*P*≤0.005).

The data presented in **Figures 5A–F** show the effects of the extract of the tested plants on the germination rate of *Chloris barbata* weed. The data in

**Figure 5A** shows that the germination rate of *Chloris barbata* decreased with increasing extract concentration of *Citrullus colocynthis*. The highest germination rate of *Chloris barbata* was observed in control followed by 5g/L, while no germination was observed at 20, 35, and 50 g/L of *Citrullus colocynthis* extracts. The same results were obtained by Mseddi et al. (2018) who found that *Citrullus colocynthis* application reduced the growth of ryegrass weed. The effect of *Citrullus colocynthis* extract on reducing the germination of *Chloris barbata* could be due to the high content of d-Glycero-d-galacto-heptose and Propane in *Citrullus colocynthis* extract (**Figure 2A**).

**Figure 5B** indicates that the germination rate of *Chloris barbata* decreased as the extract concentration of *Euphorbia retusa* increased. The control of *Chloris barbata* seed exhibited the highest germination rate followed by 5 and 20 g/L (without significant difference between them). Additionally, no germination of *Chloris barbata* was observed at 35 and 50 g/L of *Euphorbia retusa*. The effect of *Euphorbia retusa* extract on reducing the germination of *Chloris barbata* could be due to the high content of 9,19-Cyclolanost-24-en-3-ol, (3.beta.) and 9,19-Cyclolanostan-3-ol, 24-methylene-, (3.beta.) in *Euphorbia retusa* extract (**Figure 2B**).

**Figure 5C** indicates that the germination rate of *Chloris barbata* gradually decreased with increasing the extract concentration of *Retama raetam*. The highest germination rate of *Chloris barbata* was observed in the control followed by 5 and 20 g/L, while no germination was observed by using either 35 or 50 g/L. In accordance with our results, El-darier et al. (2018) found that crude powder of the aerial shoots of *Retama raetam* suppressed the germination of both *Triticum aestivum* and *Phalaris minor*. The effect of *Retama raetam* extract on reducing the germination of *Chloris barbata* might be due to the high content of Dodecahydro-7,14-methanodipyrido[1,2-a:1’,2’-e][1,5]diazocine in *Retama raetam* extract (**Figure 2C**).

According to the information presented in **Figure 5D**, the germination rate of *Chloris barbata* decreased as the extract concentration of *Artemisia monosperma* increased. The control exhibited the highest germination rate followed by 5 g/L and no growth was observed by all higher concentrations (20, 35, and 50 g/L).

The effect of *Artemisia monosperma* extract on reducing the germination of *Chloris barbata* might be due to the high content of 2-Methylamino-N-phenyl-acetamide, 17-Pentatriacontene, and gamma-Sitosterol diazocine in *Artemisia monosperma* extract (**Figure 2D**). Studies have shown that *Artemisia monosperma* produces a variety of secondary metabolites that have allelopathic properties such as camphor, cineole, and alpha-pinene (Ivănescu et al., 2021).

**Figure 5E** reveals that the germination rate of *Chloris barbata* gradually decreased with increasing extract concentration of *Tamarix gallica* extract. The control group had the highest germination rate followed by 5 g/L, while no germination was observed at higher concentrations. The effect of *Tamarix gallica* extract on reducing the germination of *Chloris barbata* might be due to the high content of Androst-5-en-17-one, 3-(acetyloxy)-19-hydroxy-, (3.beta.), p-Dimethylaminobenzylidene p-anisidine, and Benzene, 1-(1-buten-3-yl)-2-vinyl- diazocine in *Tamarix gallica* extract (**Figure 2E**). Studies have shown that *Tamarix gallica* produces a variety of allelopathic compounds, including phenolic acids, flavonoids, and terpenes (Khan et al., 2018). The data presented in

**Figure 5F** suggests that all concentrations of *Artemisia judaica* extract suppressed the germination of *Chloris barbata* compared to the control. The effect of *Artemisia judaica* extract on reducing the germination of *Chloris barbata* might be due to the high content of Naphtho[1,2-b] furan-2,6(3H,4H)-dione, 3a,5,5a,9,9a,9b-hexahydro-9-hydroxy-3,5a,9-trimethyl in *Artemisia judaica* extract (**Figure 2A**). Studies have shown that *Artemisia judaica* produces a variety of secondary metabolites that have allelopathic properties such as camphor, terpinen-4-ol, and alpha-pinene (Yosef Friedjung et al., 2013).

### 3.3 The effect of wild plant extracts on weed root growth

#### 3.3.1 Response of *Amaranthus retroflexus* seedling root to treatment with wild plant extracts

**Figure 6:**
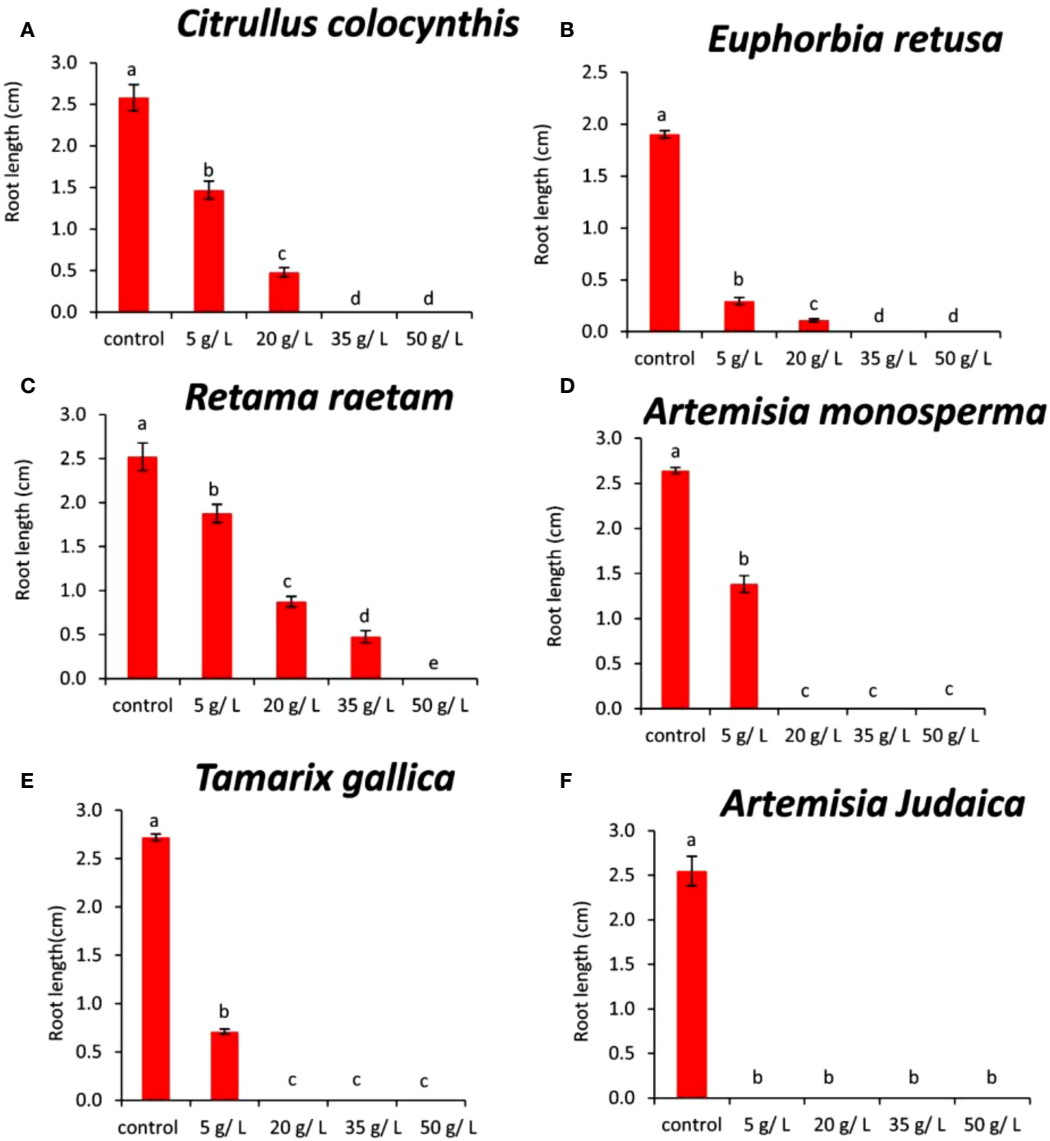
Effect of different extract concentrations of investigated wild plants on the root length of *Amaranthus retroflexus.* **(A)** *Citrullus colocynthis*, **(B)** *Euphorbia retusa*, **(C)** *Retama raetam*, **(D)** *Artemisia monosperma*, **(E)** *Tamarix gallica* and **(F)** *Artemisia judaica* extracts. Values are the mean ± SE of two biological replicates each with 30 seeds. Bars of the graphs represent standard errors. Significant differences in means are indicated by different letters according to Tukey’s test (*P*≤0.005).

The data in **Figure 6A** indicated that the root length of *Amaranthus retroflexus* decreased with increasing extract concentration of *Citrullus colocynthis*. The highest root length of *Amaranthus retroflexus* was observed in control followed by 5 and 20 mg/L, respectively. No root elongation was observed at 35 and 50 g/L of *Citrullus colocynthis*. Also, in accordance with our results, Iqbal et al. (2020) examine the herbicidal potential of aqueous extracts of *Citrullus colocynthis* fruit and vine at four concentrations (25, 50, 75, and 100%) against *Lathyrus aphaca*, a prevalent weed of wheat in the area under study. They mentioned that fruits and vines demonstrated strong herbicidal potential, suppressing several *L. aphaca* characteristics such as reduction in the number of leaves, shoot length, root length, shoot weight, root fresh weight, shoot dry weight, root dry weight, and nodule numbers. The effect of *Citrullus colocynthis* extract on the root development of *Amaranthus retroflexus* could be due to the high content of d-Glycero-d-galacto-heptose and Propane in *Citrullus colocynthis* extract (**Figure 2A**).

The information presented in **Figure 6B** suggests that the root length of *Amaranthus retroflexus* decreased as the extract concentration of *Euphorbia retusa* increased until 20 g/L and no rooting was detected at 35 and 50 g/L. The effect of *Euphorbia retusa* extract on reducing the germination of *Amaranthus retroflexus* could be due to the high content of 9,19-Cyclolanost-24-en-3-ol, (3.beta.) and 9,19-Cyclolanostan-3-ol, 24-methylene-, (3.beta.) in *Euphorbia retusa* extract (**Figure 2B**). These results are in agreement with previous results which indicated that the root growth of *Chenopodium murale* was more affected than the shoot system to the inhibitory allelopathic activity induced by Euphorbia retusa, which may attributed to being the radicle the first to emerge and consequently direct contact with the extracts (El-amier et al., 2021).

**Figure 6C** indicates that the root length of *Amaranthus retroflexus* decreased with gradually increasing extract concentration of *Retama raetam*. The highest root length of *Amaranthus retroflexus* was observed in the control and no rooting was observed at 50 mg/L.

The effect of *Retama raetam* extract on restricting the growth of *Amaranthus retroflexus* roots might be due to the high content of Dodecahydro-7,14-methanodipyrido[1,2-a:1’,2’-e][1,5]diazocine in *Retama raetam* extract (**Figure 2C**).

According to the information presented in **Figure 6D**, the root length of *Amaranthus retroflexus* decreased significantly at the concentration of 5 g/L of *Artemisia monosperma* compared to the control. There was no rooting recorded at 20, 35, and 50 g/L. The effect of *Artemisia monosperma* extract on reducing the germination of *Amaranthus retroflexus* might be due to the high content of 2-Methylamino-N-phenyl-acetamide, 17-Pentatriacontene, and gamma-Sitosterol diazocine in *Artemisia monosperma* extract (**Figure 2D**).

Additionally, the most probable explanation for the reductions in weed rooting is the reduced rate of cell division and cell elongation due to the presence of allelochemicals in the aqueous extracts of *Artemisia monosperma* (Javaid and Anjum, 2006).

**Figure 6E** reveals that the root length of *Amaranthus retroflexus* decreased significantly at the concentration 5 g/L of *Tamarix gallica* extract compared to the control. No rooting of *Amaranthus retroflexus* seed was observed when treated with 20, 35, and 50 g/L of *Tamarix gallica* extract. The effect of *Tamarix gallica* extract on reducing the germination of *Amaranthus retroflexus* might be due to the high content of Androst-5-en-17-one, 3-(acetyloxy)-19-hydroxy-, (3.beta.), p-Dimethylaminobenzylidene p-anisidine, and Benzene, 1-(1-buten-3-yl)-2-vinyl- diazocine in *Tamarix gallica* extract (**Figure 2E**). In accordance with our results, previous work found that *Tamarix gallica* produces a variety of allelopathic compounds, including phenolic acids, flavonoids, and terpenes (Khan et al., 2018).

The data presented in **Figure 6F** suggests that there was no rooting of *Amaranthus retroflexus* seeds observed with all concentrations of *Artemisia judaica* compared to the control. The effect of *Artemisia judaica* extract on reducing the germination of *Amaranthus retroflexus* might be due to the high content of Naphtho[1,2-b] furan-2,6(3H,4H)-dione, 3a,5,5a,9,9a,9b-hexahydro-9-hydroxy-3,5a,9-trimethyl in *Artemisia judaica* extract (**Figure 2A**). Studies have shown that *Artemisia judaica* produces a variety of secondary metabolites that have allelopathic properties such as camphor, terpinen-4-ol, and alpha-pinene (Yosef Friedjung et al., 2013).

#### 3.3.2 Response of *Portulaca oleracea* seedling root to wild plant extracts

**Figure 7:**
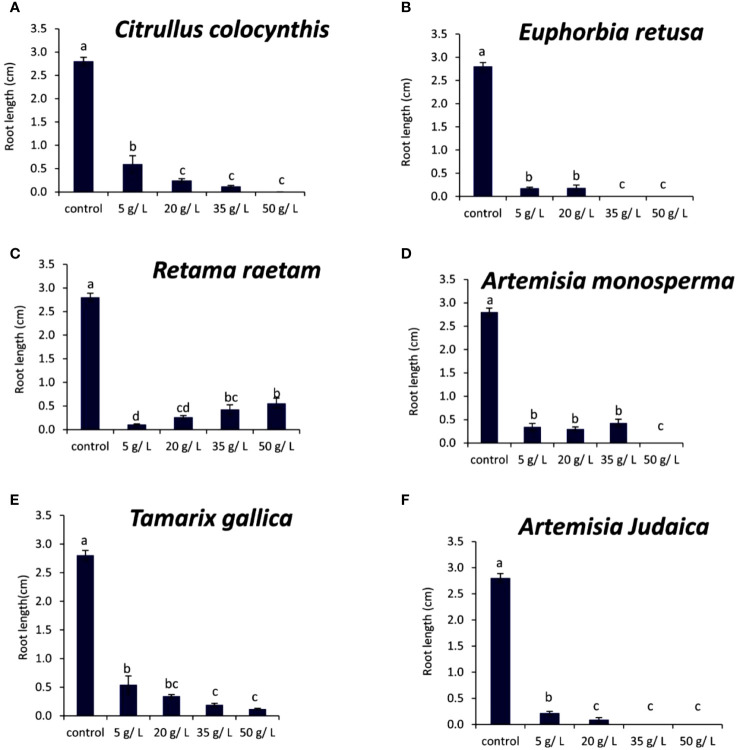
Effect of different extract concentrations of studied wild plants on the root length of *Portulaca oleracea.* **(A)** *Citrullus colocynthis*, **(B)** *Euphorbia retusa*, **(C)** *Retama raetam*, **(D)** *Artemisia monosperma*, **(E)** *Tamarix gallica* and **(F)** *Artemisia judaica* extracts. Values are the mean ± SE of two biological replicates each with 30 seeds. Bars of the graphs represent standard errors. Significant differences in means are indicated by different letters according to Tukey’s test (*P*≤0.005).

The data in **Figure 7A** indicated that the root length of *Portulaca oleracea* decreased with increasing extract concentration of *Citrullus colocynthis*. The highest root length of *Portulaca oleracea* was observed in control, while the lowest root length was observed at 20, 35, 50 g/L of *Citrullus colocynthis* without significant difference between them. The effect of *Citrullus colocynthis* extract on reducing the germination of *Portulaca oleracea* could be due to the high content of d-Glycero-d-galacto-heptose and Propane in *Citrullus colocynthis* extract (**Figure 2A**).

The results presented in **Figure 7B** showed that the root length of *Portulaca oleracea* decreased as the extract concentration of *Euphorbia retusa* increased. The control of *Portulaca oleracea* seed exhibited the highest root length compared to the other treatments. However, no significant difference was observed in the root length of *Portulaca oleracea* at 5 and 20 g/L of *Euphorbia retusa*. Additionally, there was no rooting recorded at 35 and 50 g/L. The effect of *Euphorbia retusa* extract on reducing the germination of *Portulaca oleracea* could be due to the high content of 9,19-Cyclolanost-24-en-3-ol, (3.beta.) and 9,19-Cyclolanostan-3-ol, 24-methylene-, (3.beta.) in *Euphorbia retusa* extract (**Figure 2B**).

**Figure 7C** indicates that the root length of *Portulaca oleracea* decreased with *Retama raetam* application. The highest root length of *Portulaca oleracea* was observed in the control. No significant difference was recorded in root length between 5 and 20 g/L and between 35 and 50 g/L. The effect of *Retama raetam* extract on reducing the germination of *Portulaca oleracea* might be due to the high content of Dodecahydro-7,14-methanodipyrido[1,2-a:1’,2’-e][1,5]diazocine in *Retama raetam* extract (**Figure 2C**).

In **Figure 7D**, the root length of Portulaca oleracea significantly decreased when the extracts of *Artemisia monosperma* were applied at concentrations 5, 20, and 35 g/L as compared to the control (without significant difference between them). The 50 g/L treatment significantly exhibited the lowest root length compared to other treatments. The effect of *Artemisia monosperma* extract on reducing the germination of *Portulaca oleracea* might be due to the high content of 2-Methylamino-N-phenyl-acetamide, 17-Pentatriacontene, and gamma-Sitosterol diazocine in *Artemisia monosperma* extract (**Figure 2D**). Furthermore, allelochemicals of *Artemisia monosperma* could inhibit the elongation, expansion and division of cells which were a prerequisite for growth of roots (Einhellig, 1995).

**Figure 7E** showed that the root length of *Portulaca oleracea* gradually decreased with increasing *Tamarix gallica* extract concentration from 5 to 50 g/L. The control seedlings had the highest root length, while 35 and 50 g/L of *Tamarix gallica* extract showed a lower root length.

The effect of *Tamarix gallica* extract on reducing the germination of *Portulaca oleracea* might be due to the high content of Androst-5-en-17-one, 3-(acetyloxy)-19-hydroxy-, (3.beta.), p-Dimethylaminobenzylidene p-anisidine, and Benzene, 1-(1-buten-3-yl)-2-vinyl- diazocine in *Tamarix gallica* extract (**Figure 2E**). Studies have shown that *Tamarix gallica* produces a variety of allelopathic compounds, including phenolic acids, flavonoids, and terpenes. (Khan et al., 2018). Also, several studies had shown that compounds of plant origin, such as allelochemicals, affect mitotic activity of growing roots (Rizvi et al., 1992; Einhellig, 1995).

The data in **Figure 7F** suggests that the root length of *Portulaca oleracea* decreased with increasing extract concentration of *Artemisia judaica* extract while there was no rooting observed in *Portulaca oleracea* seeds at the rate of 35 and 50 g/L of *Artemisia judaica* extracts. The effect of *Artemisia judaica* extract on reducing the germination of *Portulaca oleracea* might be due to the high content of Naphtho [1,2-b] furan-2,6(3H,4H)-dione, 3a,5,5a,9,9a,9b-hexahydro-9-hydroxy-3,5a,9-trimethyl in *Artemisia judaica* extract (**Figure 2A**). The previous results could be due to an inhibitory effect on mitosis that directly decreases plant growth. So mitotic activity can be used to evaluate root growth resulting from cell division of meristematic cells and cell expansion in the elongation zone of roots (Dayan et al., 2000).

#### 3.3.3 Response of *Chloris barbata* seedling root to wild plant extracts

**Figure 8:**
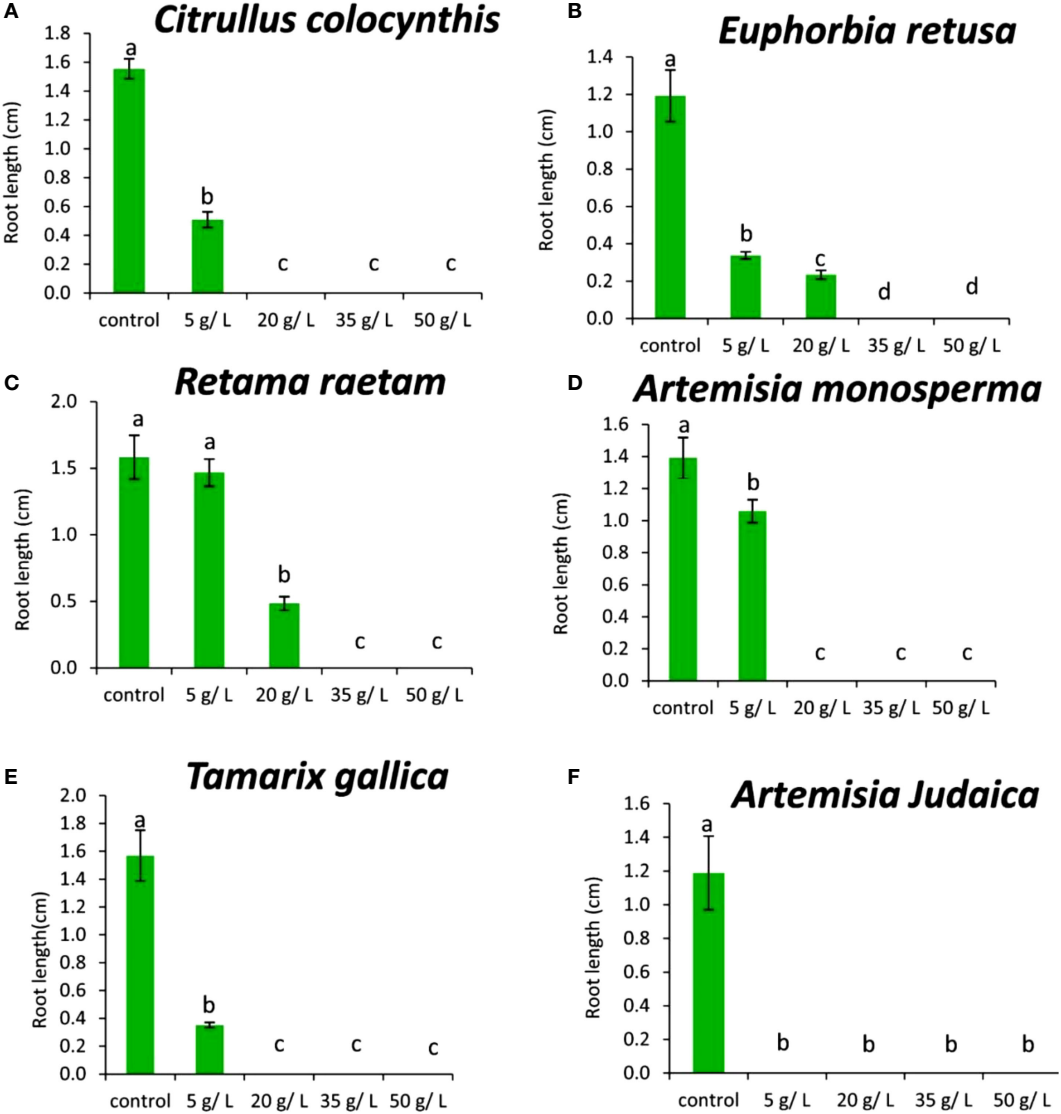
Effect of different extract concentrations of tested wild plants on the root length of *Chloris barbata.* **(A)** Citrullus colocynthis, **(B)** *Euphorbia retusa*, **(C)** *Retama raetam*, **(D)** *Artemisia monosperma*, **(E)** *Tamarix gallica* and **(F)** *Artemisia judaica* extracts. Values are the mean ± SE of two biological replicates each with 30 seeds. Bars of the graphs represent standard errors. Significant differences in means are indicated by different letters according to Tukey’s test (*P*≤0.005).

**Figure 8A** shows that the root length of *Chloris barbata* was decreased by 5 g/L of *Citrullus colocynthis* extract compared to the control. Additionally, there was no rooting observed at 20, 35, or 50 g/L of *Citrullus colocynthis*. The effect of *Citrullus colocynthis* extract on reducing the germination of *Chloris barbata* could be due to the high content of d-Glycero-d-galacto-heptose and Propane in *Citrullus colocynthis* extract (**Figure 2A**).

The information present data in **Figure 8B** showed that the root length of *Chloris barbata* decreased as the extract concentration of *Euphorbia retusa* increased. The control treatment exhibited the highest root length followed by 5 and 20 g/L, respectively. Additionally, there was no rooting was recorded at 35 and 50 g/L. The effect of *Euphorbia retusa* extract on reducing the germination of *Chloris barbata* could be due to the high content of 9,19-Cyclolanost-24-en-3-ol, (3.beta.) and 9,19-Cyclolanostan-3-ol, 24-methylene-, (3.beta.) in *Euphorbia retusa* extract (**Figure 2B**).

**Figure 8C** indicates that there was no significant difference in the root length of *Chloris barbata* between 5 g/L of *Retama raetam* and the control while 20 g/L significantly decreased the root length of *Chloris barbata.* Also, there was no rooting observed at 35 and 50 g/L. The effect of *Retama raetam* extract on reducing the germination of *Chloris barbata* might be due to the high content of Dodecahydro-7,14-methanodipyrido[1,2-a:1’,2’-e][1,5]diazocine in *Retama raetam* extract (**Figure 2C**).

According to **Figure 8D**, the root length of *Chloris barbata* decreased by 5 g/L of *Artemisia monosperma* extract compared to the control. There was no rooting was observed at 20, 35, 50 g/L. The effect of *Artemisia monosperma* extract on reducing the germination of *Chloris barbata* might be due to the high content of 2-Methylamino-N-phenyl-acetamide, 17-Pentatriacontene, and gamma-Sitosterol diazocine in *Artemisia monosperma* extract (**Figure 2D**). The most probable explanation for the reductions in seedling root growth is the reduced rate of cell division and cell elongation due to the presence of allelochemicals in the aqueous extracts (Javaid and Anjum, 2006).

**Figure 8E** showed that the root length of *Chloris barbata* decreased significantly by extract 5 g/L of *Tamarix gallica*. Also, 20, 35 and 50 g/L of *Tamarix gallica* extract showed no rooting. The effect of *Tamarix gallica* extract on reducing the germination of *Chloris barbata* might be due to the high content of Androst-5-en-17-one, 3-(acetyloxy)-19-hydroxy-, (3.beta.), p-Dimethylaminobenzylidene p-anisidine, and Benzene, 1-(1-buten-3-yl)-2-vinyl- diazocine in *Tamarix gallica* extract (**Figure 2E**).

Our results are in agreement with Einhellig (1995) who mentioned that allelochemicals could inhibit the elongation, expansion and division of cells that are prerequisites for growth. Additionally, studies have shown that *Tamarix gallica* produces a variety of allelopathic compounds, including phenolic acids, flavonoids, and terpenes (Khan et al., 2018).

The data in **Figure 8F** show that all *Artemisia judaica* concentrations tested in the present work inhibited the rooting of *Chloris barbata* compared to the control. The effect of *Artemisia judaica* extract on reducing the germination of *Chloris barbata* might be due to the high content of Naphtho[1,2-b] furan-2,6(3H,4H)-dione, 3a,5,5a,9,9a,9b-hexahydro-9-hydroxy-3,5a,9-trimethyl in *Artemisia judaica* extract (**Figure 2A**). Several studies had shown that compounds of plant origin, such as allelochemicals, affect mitotic activity of growing roots (Rizvi et al., 1992, Einhellig, 1995).

### 3.4 The effect of wild plant extracts on weed plumule growth

#### 3.4.1 Response of *Amaranthus retroflexus* seedling plumule to treatment with different concentrations of wild plant extracts

**Figure 9:**
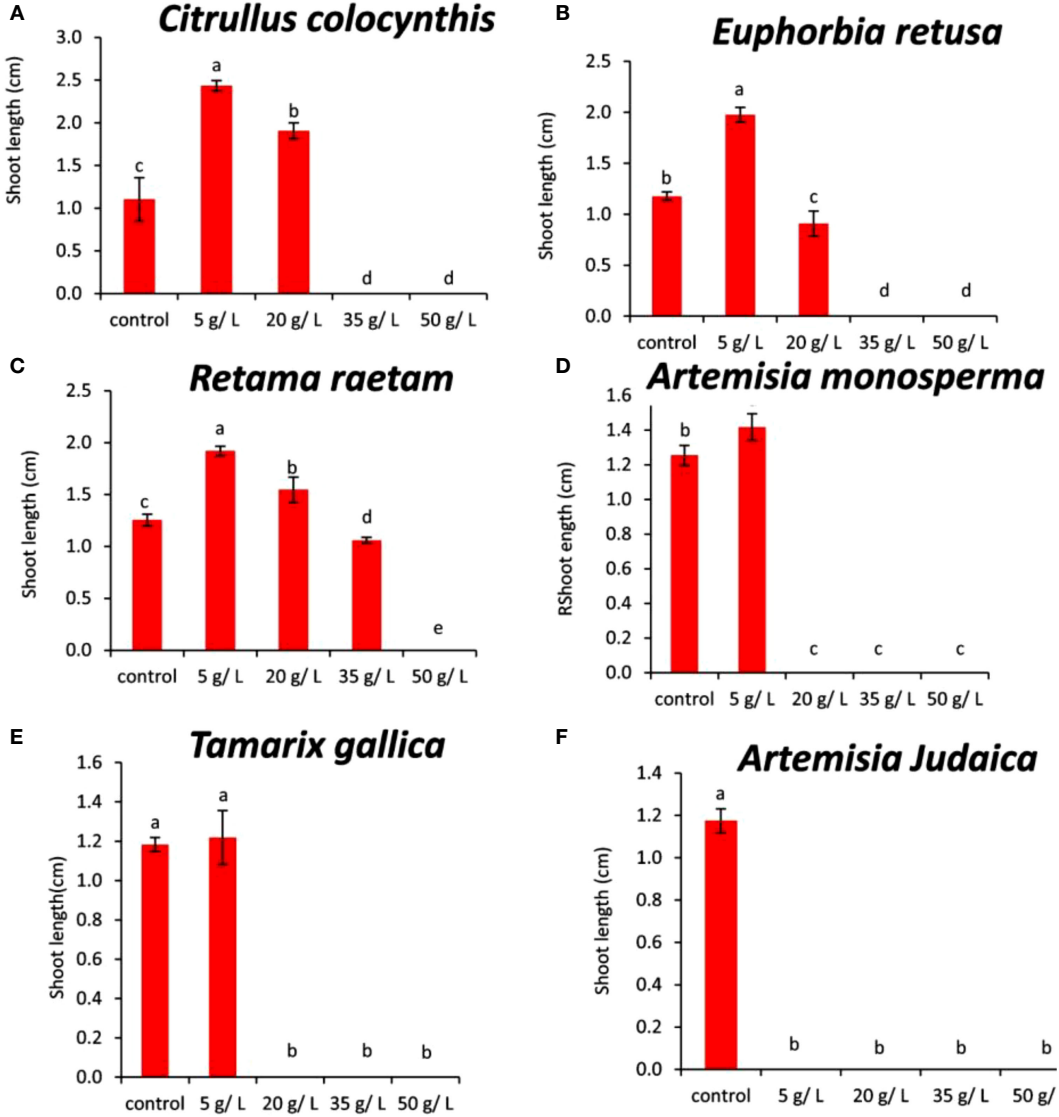
Effect of different extract concentrations of tested wild plants on the shoot length of *Amaranthus retroflexus.* **(A)** *Citrullus colocynthis*, **(B)** *Euphorbia retusa*, **(C)** *Retama raetam*, **(D)** *Artemisia monosperma*, **(E)** *Tamarix gallica* and **(F)** *Artemisia judaica* extracts. Values are the mean ± SE of two biological replicates each with 30 seeds. Bars of the graphs represent standard errors. Significant differences in means are indicated by different letters according to Tukey’s test (*P*≤0.005).

Data showed in **Figure 9A** indicated that the shoot length of *Amaranthus retroflexus* increased at the concentration of 5 and 20 g/L of *Citrullus colocynthis* extract compared to the control. Additionally, the inhibition of the shoot growth of *Amaranthus retroflexus* was observed at 35 and 50 g/L of *Citrullus colocynthis*. Also, Iqbal et al. (2020) found that *Citrullus colocynthis* fruit and vine extracts at four concentrations (25, 50, 75, and 100%) had a strong effect in minimizing the growth of *Lathyrus aphaca*. They mentioned that fruits and vines demonstrated strong herbicidal potential, suppressing many of leaves, shoot length, root length, shoot fresh weight, root fresh weight, shoot dry weight, root dry weight, and nodule numbers. According to data presented in **Figure 9B**, the shoot length of *Amaranthus retroflexus* was higher when the applied *Euphorbia retusa* extract concentration was 5 g/L compared to the control. However, the concentration of 20 g/L showed lower shoot growth compared to the control. Moreover, no shoot growth was observed when treated with *Euphorbia retusa* extracts at the concentrations of 35 and 50 g/L. **Figure 9C** indicated that the shoot length of *Amaranthus retroflexus* increased at a rate of 5 g/L *Retama raetam* compared to the control and then decreased gradually until 35 g/L. Additionally, no growth of *Amaranthus retroflexus* was recorded at 50 g/L. According to the information presented in **Figure 9D**, 5 g/L of *Artemisia monosperma* treatment significantly increased the shoot length of *Amaranthus retroflexus* compared to the control. On the other hand, there was no shoot growth recorded at 20, 35, and 50 g/L. Such an inhibitory effect at the high rate of *Artemisia monosperma* could be due to the suppression of cell division of meristematic cells and cell expansion in the elongation zone of roots (Dayan et al., 2000). **Figure 9E** reveals that the shoot length of *Amaranthus retroflexus* increased at the concentration of 5 g/L of *Tamarix gallica* extract compared to the control but the difference was not significant. No shoot growth was detected in germinated *Amaranthus retroflexus* seeds when treated with 20, 35, and 50 g/L of *Tamarix gallica* extract.

Allelochemicals from *Tamarix gallica* may be responsible for the inhibitory effects on shoot development by preventing cell division, elongation, and expansion that are necessary for growth (Einhellig, 1995). The data presented in **Figure 9F** shows that treatment with *Artemisia judaica* at all investigated concentrations inhibited *Amaranthus retroflexus* shoot growth in all tested seeds as compared to the control. Previous studies have shown that *Artemisia judaica* produces various secondary metabolites with allelopathic properties, such as camphor, terpinen-4-ol, and alpha-pinene (Yosef Friedjung et al., 2013). Additionally, the most probable explanation for the reductions in seedling root and shoot is the reduced rate of cell division and cell elongation due to allelochemicals in the aqueous extracts (Javaid and Anjum, 2006).

#### 3.4.2 Response of *Portulaca oleracea* seedling plumule to treatment with different concentrations of wild plant extracts

**Figure 10:**
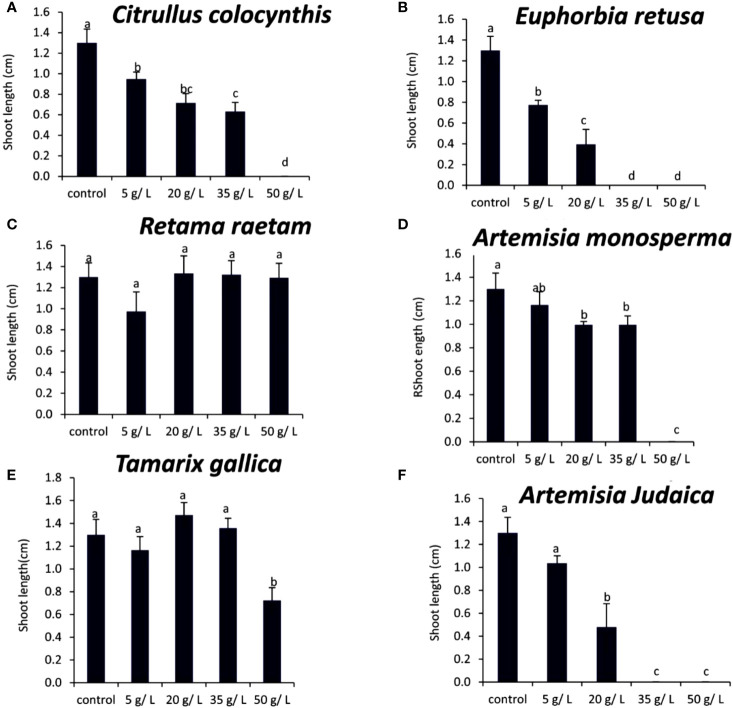
Effect of different extract concentrations of studied wild plants on the shoot length of *Portulaca oleracea.* **(A)** *Citrullus colocynthis*, **(B)** *Euphorbia retusa*, **(C)** *Retama raetam*, **(D)** *Artemisia monosperma*, **(E)** *Tamarix gallica* and **(F)** *Artemisia judaica* extracts. Values are the mean ± SE of two biological replicates each with 30 seeds. Bars of the graphs represent standard errors. Significant differences in means are indicated by different letters according to Tukey’s test (*P*≤0.005).

The results in **Figure 10A** showed that the shoot length of *Portulaca oleracea* decreased gradually with increasing extract concentration of *Citrullus colocynthis*. The highest shoot length of *Portulaca oleracea* was observed in control, while no shoot was observed at 50 g/L of *Citrullus colocynthis*. The current data in **Figure 10B** showed that the shoot length of *Portulaca oleracea* decreased as the extract concentration of *Euphorbia retusa* increased. The control of *Portulaca oleracea* seed exhibited the highest shoot length compared to the other treatments. Additionally, there was no shoot growth was recorded at 35 and 50 g/L. **Figure 10C** indicates that the shoot length of *Portulaca oleracea* was not significantly affected by any of the *Retama raetam* concentrations. According to the presented data in **Figure 10D**, the shoot length of *Portulaca oleracea* subjected to a concentration of 5 g/L of *Artemisia monosperma* did not show a significant difference from control seedlings. However, the higher concentrations (20 and 35 g/L) showed a significantly shorter plumule than other treatments. Moreover, there was no shoot growth recorded at 50 g/L.

**Figure 10E** showed that 50 g/L of *Tamarix gallica* extract was the only treatment that led to a significant decrease in *Portulaca oleracea* shoot length compared to the control and other treatments. In this regard, several studies had shown that compounds of plant origin, such as allelochemicals, affect mitotic activity of vegetative growing (Rizvi et al., 1992; Einhellig, 1995). The data shown in **Figure 10F** suggests that the shoot length of *Portulaca oleracea* decreased with increasing *Artemisia judaica* extract concentration. Still, this reduction in shoot length was insignificant when the treatment concentration was applied at 5 g/L, while it was evident when the treatment was conducted at 20 mg/L of *Artemisia judaica* extract. There was no shoot produced by *Portulaca oleracea* seeds at the rate of 35 and 50 g/L of *Artemisia judaica*. Studies have shown that *Artemisia judaica* produces a variety of secondary metabolites that have allelopathic properties such as camphor, terpinen-4-ol, and alpha-pinene (Yosef Friedjung et al., 2013). Furthermore, allelochemicals could inhibit the elongation, expansion and division of cells which were a prerequisite for growth (Einhellig, 1995).

#### 3.4.3 Response of Chloris barbata seedling plumule to treatment with different concentrations of wild plant extracts

**Figure 11:**
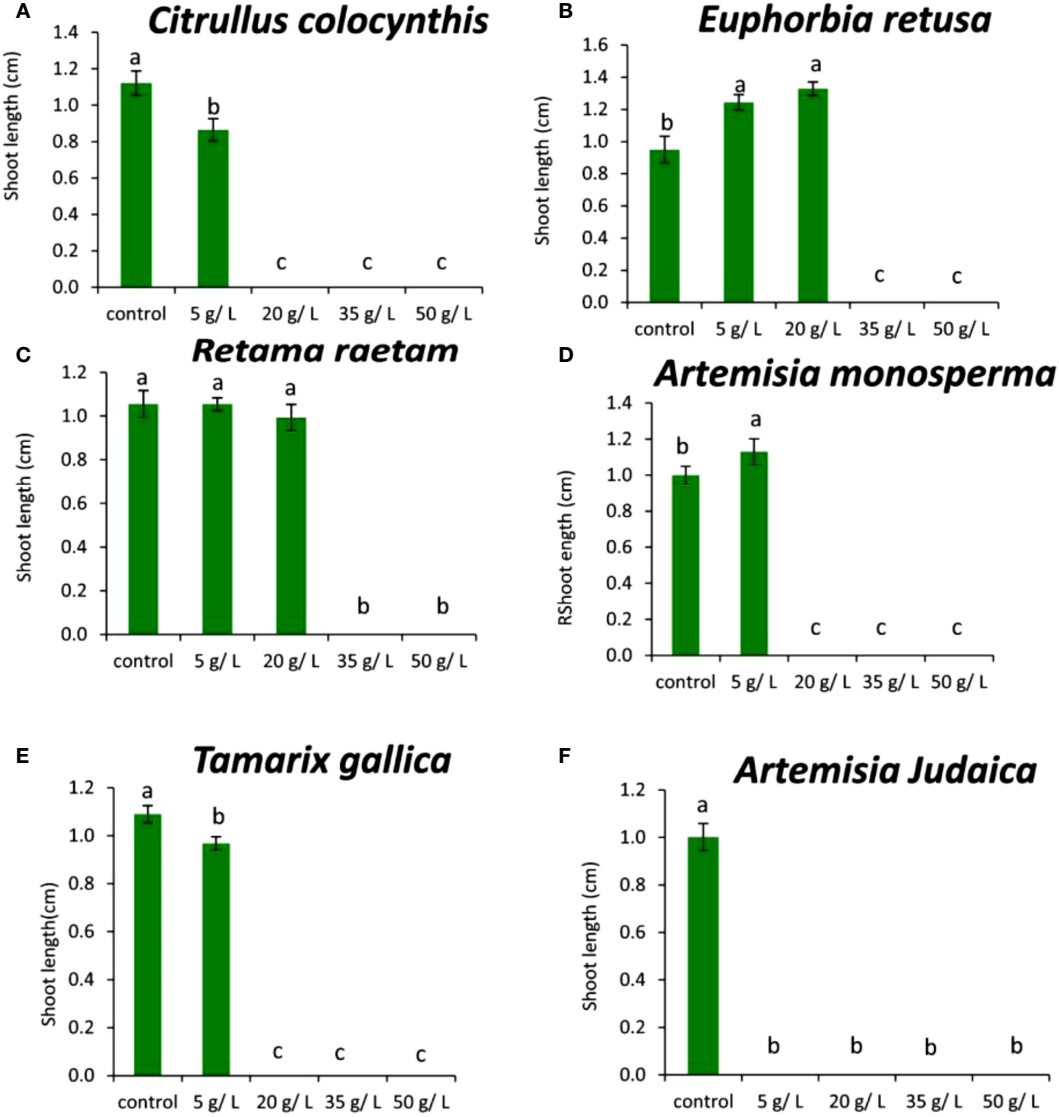
Effect of different extract concentrations of studied wild plants on the shoot length of *Chloris barbata.* **(A)** *Citrullus colocynthis*, **(B)** *Euphorbia retusa*, **(C)** *Retama raetam*, **(D)** *Artemisia monosperma*, **(E)** *Tamarix gallica* and **(F)** *Artemisia judaica* extracts. Values are the mean ± SE of two biological replicates each with 30 seeds. Bars of the graphs represent standard errors. Significant differences in means are indicated by different letters according to Tukey’s test (*P*≤0.005).

The results in **Figure 11A** showed that the shoot length of *Chloris barbata* decreased by the application of 5 g/L *Citrullus colocynthis* extract. The highest shoot length of *Chloris barbata* was observed in control, while no shoot was observed at 20, 35, and 50 g/L of *Citrullus colocynthis*.

The current data in **Figure 11B** showed that the shoot length of *Chloris barbata* increased by 5 and 20 g/L application with the extract of *Euphorbia retusa* compared to the control. However, there was no shoot growth was recorded at 35 and 50 g/L.

**Figure 11C** indicates that the shoot length of *Chloris barbata* was not significantly affected by 5 and 20 g/L of the *Retama raetam* compared to the control. However, there was no shoot growth recorded at 35 and 50 g/L.

The data in **Figure 11D** indicated that the shoot length of *Chloris barbata* at the 5 g/L of *Artemisia monosperma* significantly increase shoot length compared to the control. However, the higher concentrations (20, 35, and 50 g/L) completely suppressed the shoot growth of *Chloris barbata*.

**Figure 11E** showed that 5 g/L of *Tamarix gallica* extract negatively affected *Chloris barbata* shoot length and this reduction was significant compared to the control but clearly higher than applications at other treatment concentrations (20, 35, and 50 g/L) which completely suppressed *Chloris barbata* shoot growth.

The data in **Figure 11F** show that all concentrations of *Artemisia judaica* extracts inhibited *Chloris barbata* radicle protrusion, thus, plumule emergence in all tested seeds compared to the control. The inhibitory effect of *Artemisia judaica* could be due to its role for inhibiting cell division of meristematic cells and cell expansion (Dayan et al., 2000).

### 3.5 Weed morphology in response to treatments with different concentrations of studied wild plants extracts

#### 3.5.1 Amaranthus retroflexus seedling morphology in response to treatment with different concentrations of wild plant extracts

Five plant extract concentrations (0, 5, 20, 35, and 50 g L^-1^) were investigated to assess the impact of selected wild plants from the Tabuk Region on suppressing the growth and development of *Amaranthus retroflexus* seedlings.

**Figure 12:**
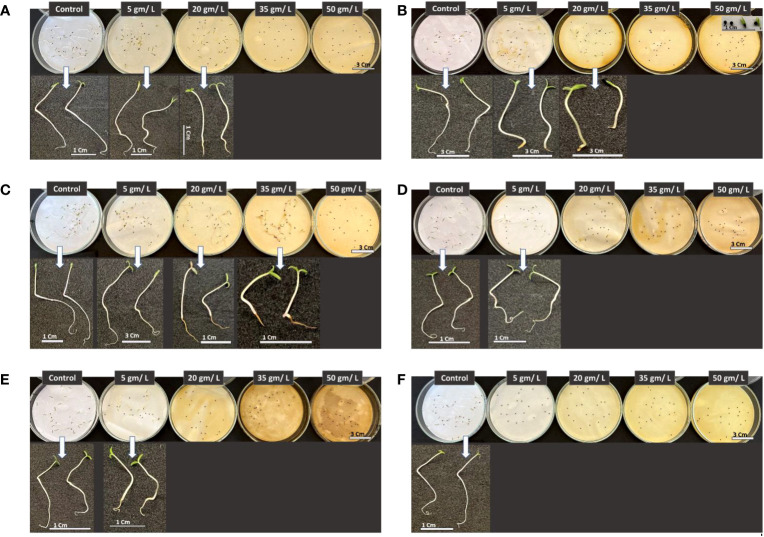
Phenotype of *Amaranthus retroflexus* seedlings in response to treatments with different wild plant extracts at different studied concentrations (Control, 5, 20, 35, and 50 mg/L). Photographs were taken on day eight post seed sowing. Effect of different investigated plant aquatic extract concentrations of **(A)** *Citrullus colocynthis*, **(B)** *Euphorbia retusa*, **(C)** *Retama raetam*, **(D)** *Artemisia monosperma*, **(E)** *Tamarix gallica* and **(F)** *Artemisia judaica* on *A. retroflexus* seedling growth and development.

**Figure 12** illustrates a clear suppression of the weed *Amaranthus retroflexus* seed germination along with inhibitory effect on seedling development, however, this impact varies depending on plant extract and concentration. In all cases, roots were the most affected plant part by different treatments. When comparing different plant extract impact on the weed germination and growth, it can be seen the apparent repression caused by the the higher levels of extracts at 35 and 50 g/L except for Retama raetam extract which allowed weed seeds to germinate but seedlings were shorter compared with other treatments at lower concentrations and control. It has been demonstrated that the extract of plants with allelopathic effect can significantly inhibit root growth and disturb the morphological differentiation of susceptible plants (Chon et al., 2002). In general, the results revealed that studied wild plants possess the ability to affect assessed weedy plants which can be attributed to their ability in creating secondary metabolites that have substantial bioactivity.

#### 3.5.2 *Portulaca oleracea* seedling morphology in response to treatment with different concentrations of wild plant extracts

**Figure 13:**
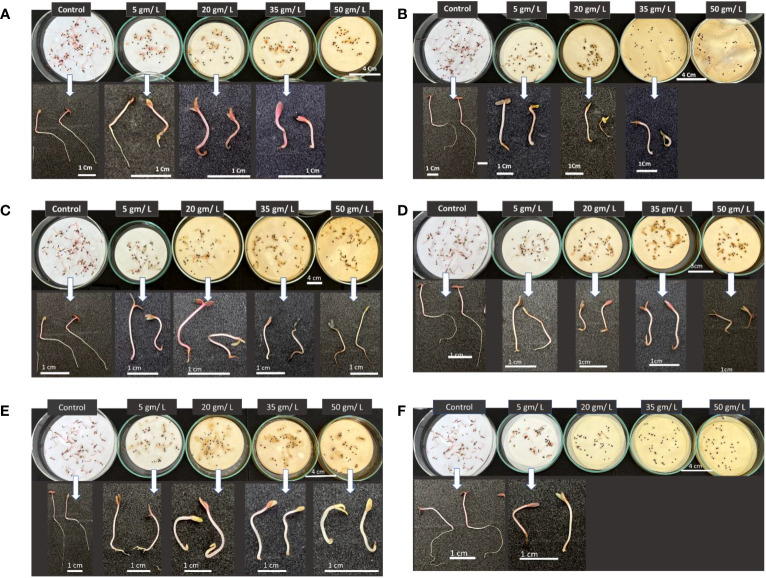
Phenotype of *Portulaca oleracea* seedlings in response to treatments with different wild plant extracts at different tested concentrations (Control, 5, 20, 35, and 50 mg/L). Photographs were taken on day eight post seed sowing. Effect of different investigated plant aquatic extract concentrations of **(A)** *Citrullus colocynthis*, **(B)** *Euphorbia retusa*, **(C)** *Retama raetam*, **(D)** *Artemisia monosperma*, **(E)** *Tamarix gallica* and **(F)** *Artemisia judaica* on *Portulaca oleracea* seedling growth and development.

**Figure 13** shows the morphological traits of *Portulaca oleracea* in response to applying wild plant extracts at different concentrations. Purslane seed germination was significantly negatively affected by treatments with extracts of different studied plants at all tested concentrations. Germinated purslane seedlings showed abnormality in their growth and development. This result can be attributed to changes or imbalance in the composition of phytohormones which prevents plants from growing and developing normally (Cheng and Cheng, 2015).

#### 3.5.3 *Chloris barbata* seedling morphology in response to treatment with different concentrations of wild plant extracts

**Figure 14:**
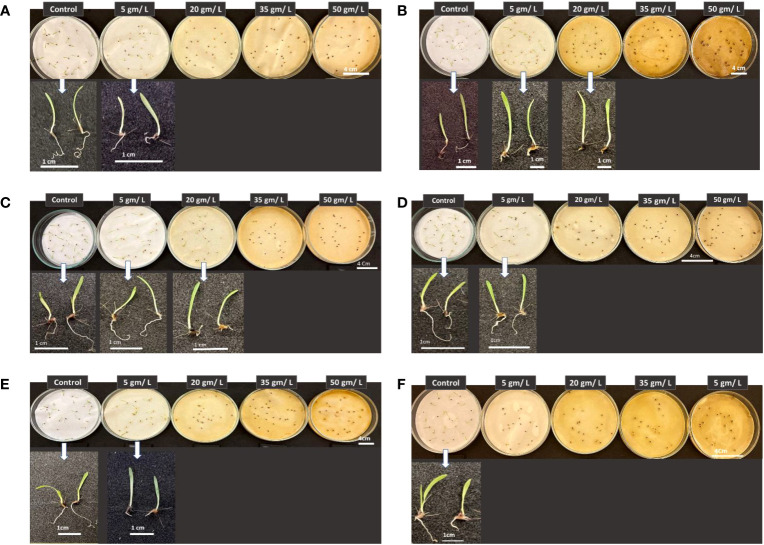
Phenotype of *Chloris barbata* seedlings in response to treatments with different wild plant extracts at different investigated concentrations (Control, 5, 20, 35, and 50 mg/L). Photographs were taken on day eight post seed sowing. Effect of different investigated plant aquatic extract concentrations of **(A)** Citrullus colocynthis, **(B)** Euphorbia retusa, **(C)** Retama raetam, **(D)** Artemisia monosperma, **(E)** Tamarix gallica and **(F)** *Artemisia judaica* on Chloris barbata seedling growth and development.

Results presented in **Figure 14** show the effect of treatment with the five tested concentrations in the present work of the selected wild plants on *Chloris barbata* seedling development. The latter significantly inhibited *C. barbata* seed germination and thus the growth of the embryo at all investigated concentrations **Figure 14F**. On the other hand, the rest of the wild plants tested herein vary in their impact on *C. barbata* seedling growth, but in all cases the higher concentrations (35 and 50 g/L) were efficient in suppressing seed germination of the weedy plant *C. barbata* and thus the growth of the miniature plant. It can be observed that all plant extracts at all studied concentrations affected *C. barbata* root morphological traits.

## 4 Conclusion

The current study evaluates the extract of six wild plants from the Tabuk Region namely *Citrullus colocynthis*, *Retama raetam*, *Euphorbia retusa*, *Artemisia monosperma*, *Tamarix gallica* and *Artemisia judaica* at four different concentrations (5, 20, 35, and 50 g/L) for controlling three weeds’ species including *Amaranthus retroflexus*, *Portulaca oleracea, and Chloris barbata* compared to the control treatments using distilled water. The results indicated that all tested species had a strong suppression effect on the germination and growth of the three tested weed species. The most effective rates in all applied wild plant extracts were 35 and 50 g/L. The low rates (5 and 15 g/L) had low effects compared to the high concentrations. The present results provided strong evidence that investigated wild plants have significant allelopathic potential that varies from one plant to another and from one weed to another. The highest allelopathic potential was shown by *Artemisia judaica* at all tested extract concentrations, while the lowest one was illustrated by *Retama raetam*. Results of GC-MS analysis of the tested plants revealed that extracts from aerial parts of the tested wild plants were rich in alkaloids, steroids, phenols, and other allelochemical compounds. These bioactive compounds lead to different levels of germination and seedling development suppression in the studied monocotyledonous weed plant *Chloris barbata* and the two assessed dicotyledonous weedy plants *Amaranthus retroflexus* and *Portulaca oleracea*, indicating that these weedy plants vary in their tolerance to the applied aqueous extracts depending on treatment concentration. Based on the results of this work, it can be concluded that growing cultivars of allelopathic plants may become a significant weed control strategy using plant-based natural extracts, especially when used in conjunction with integrated weed management.

## Data availability statement

The original contributions presented in the study are included in the article/**Supplementary Material**. Further inquiries can be directed to the corresponding author.

## Author contributions

BA: Conceptualization, Data curation, Formal analysis, Investigation, Methodology, Project administration, Resources, Software, Supervision, Validation, Visualization, Writing – original draft, Writing – review & editing. AA: Data curation, Investigation, Methodology, Resources, Writing – review & editing. EASA: Conceptualization, Investigation, Resources, Visualization, Writing – review & editing. RA: Conceptualization, Data curation, Investigation, Validation, Writing – review & editing. EAHA: Conceptualization, Data curation, Investigation, Resources, Writing – original draft. ADA: Conceptualization, Formal analysis, Methodology, Visualization, Writing – original draft. HA: Conceptualization, Data curation, Methodology, Resources, Writing – original draft. QA: Investigation, Methodology, Resources.
