# Supplementary material for: The Bio-herbicidal potential of some wild plants with allelopathic effects from Tabuk Region on selected local weed species
Source: Front Plant Sci. 2023 Dec 5;14:1286105. doi: 10.3389/fpls.2023.1286105 (PMC10739508; doi:10.3389/fpls.2023.1286105)
Supplement: Supplementary file 1 [file Table_1.docx]

Supplementary Material

**Sub-Table 1:** The most important bioactive identified phytochemical compounds detected in the wild plant *Citrullus colocynthis.* The plant was collected from the Tabuk Region between December 2022 and January 2023. Chemical screening of the entire aerial parts of the plant was performed utilizing GC-MS analysis. RT; retention time, MW; molecular weight, comp.; compound.

| **Plant Species** | **Total comp.** | **Most Bioactive Identified Components** | **Rt min** | **Score %** | **MW** | **Mol. Formula** | **Structure** |
| --- | --- | --- | --- | --- | --- | --- | --- |
| *Citrullus colocynthis* | 49 | Dipentaerythritol  (Polyol, Alcohol derivative) | 14.395 | 1.276 | 254.137 | [C_10_H_22_O_7_](https://pubchem.ncbi.nlm.nih.gov/#query=C10H22O7) | 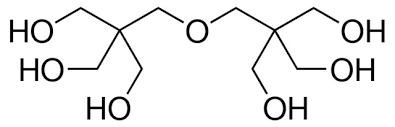 |
|  |  | Carbamic acid, ethylnitroso-, ethyl ester  (Ester Derivative) | 18.536 | 1.136 | 146.069 | [C_5_H_10_N_2_O_3_](https://pubchem.ncbi.nlm.nih.gov/#query=C5H10N2O3) |  |
|  |  | 1-Nitro-1-deoxy-d-glycero-l-mannoheptitol  (Alcohol Derivative) | 22.136 | 2.575 | 241.08 | [C_7_H_15_NO_8_](https://pubchem.ncbi.nlm.nih.gov/#query=C7H15NO8) | 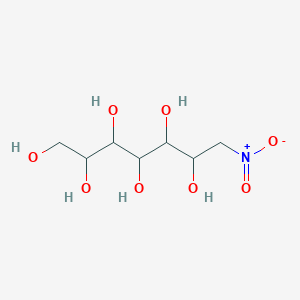 |
|  |  | 3-Aminopyrrolidine  (Alkaloid Derivative) | 23.032 | 1.846 | 86.084 | [C_4_H_10_N_2_](https://pubchem.ncbi.nlm.nih.gov/#query=C4H10N2) | 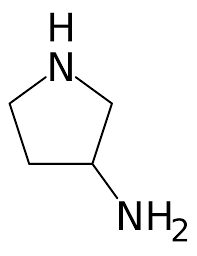 |
|  |  | Isosorbide Dinitrate  (Bifuran nitrate Derivative) | 23.734 | 0.511 | 236.028 | [C_6_H_8_N_2_O_8_](https://pubchem.ncbi.nlm.nih.gov/#query=C6H8N2O8) | 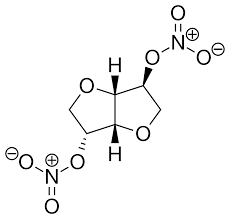 |
|  |  | (1R,2S,8As)-8-oxo-1-carboxymethyl-1,2,5,5-tetramethyl-trans-decalin  (Carboxylic acid Derivative) | 33.532 | 0.521 | 266.188 | [C_16_H_26_O_3_](https://pubchem.ncbi.nlm.nih.gov/#query=C16H26O3) |  |
|  |  | 3,5-Dimethylbenzaldehyde thiocarbamoylhydrazone  (Amine Derivative) | 35.22 | 0.732 | 207.083 | [C_10_H_13_N_3_S](https://pubchem.ncbi.nlm.nih.gov/#query=C10H13N3S) | 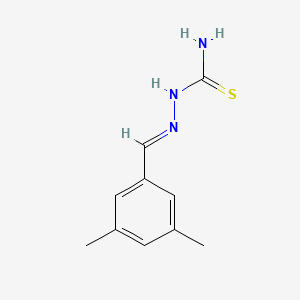 |
|  |  | 1,2,5-Oxadiazol-3-amine, 4-(4-methoxyphenoxy)-  (Amine Derivative) | 35.755 | 0.63 | 207.064 | [C_9_H_9_N_3_O_3_](https://pubchem.ncbi.nlm.nih.gov/#query=C9H9N3O3) | 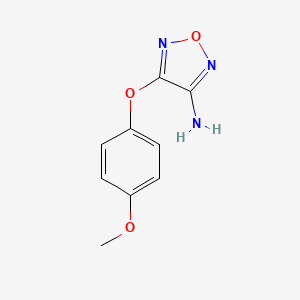 |
|  |  | 1H-1,2,4-Triazole-5(4H)-thione, 4-allyl-3-(3-furyl)-  (Thiophene Derivative) | 36.902 | 2.352 | 207.047 | [C_9_H_9_N_3_OS](https://pubchem.ncbi.nlm.nih.gov/#query=C9H9N3OS) | 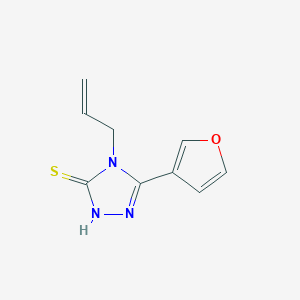 |
|  |  | dl-.alpha.-Tocopherol  (Phenolic compound Derivative) | 37.534 | 1.67 | 430.381 | [C_29_H_50_O_2_](https://pubchem.ncbi.nlm.nih.gov/#query=C29H50O2) | 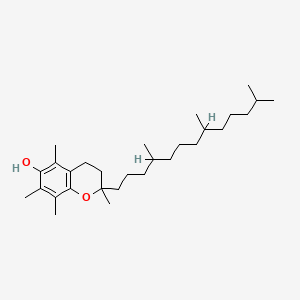 |
|  |  | Stigmasta-7,25-dien-3-ol, (3.beta.,5.alpha.)-  (Steroid Derivative) | 39.188 | 4.998 | 412.371 | [C_29_H_48_O](https://pubchem.ncbi.nlm.nih.gov/#query=C29H48O) | 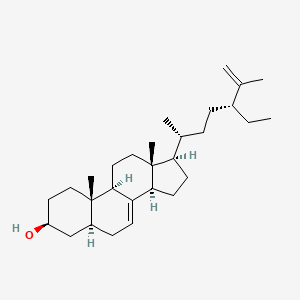 |
|  |  | Pyridine-4-carboxylic acid, 2,6-dichloro-, [5-(4-chlorophenyl)-2-thienylmethyl] ester  (Ester Derivative) | 39.688 | 6.24 | 396.95 | [C_17_H_10_Cl_3_NO_2_S](https://pubchem.ncbi.nlm.nih.gov/#query=C17H10Cl3NO2S) |  |

**Table 2:** The most important bioactive identified phytochemical compounds detected in the wild plant *Euphorbia retusa*.

The plant was collected from the Tabuk Region between December 2022 and January 2023. Chemical screening of the entire aerial parts of the plant was performed utilizing GC-MS analysis. RT; retention time, MW; molecular weight, comp.; compound.

| **Plant Species** | **Total comp.** | **Most Bioactive Identified Components** | **Rt min** | **Score %** | **MW** | **Mol. Formula** | **Structure** |
| --- | --- | --- | --- | --- | --- | --- | --- |
| *Euphorbia retusa* | 38 | Phytol ((5*R*,9*R*)-5,6,7,8,9,10,11,12-Octahydro-1,6-secoretinol)  (Alcohol derivative) | 26.701 | 0.554 | 296.308 | [C_20_H_40_O](https://pubchem.ncbi.nlm.nih.gov/#query=C20H40O) | 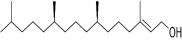 |
|  |  | Hexahydropyridine, 1-methyl-4-[4,5-dihydroxyphenyl]-  (Alkaloid derivative) (Can also be regarded as Phenolic compound derivative) | 35.734 | 0.266 | 207.126 | [C_12_H_17_NO_2_](https://pubchem.ncbi.nlm.nih.gov/#query=C12H17NO2) |  |
|  |  | Dodecahydropyrido[1,2-b]isoquinolin-6-one  (Ketone derivative) | 36.311 | 0.22 | 207.162 | [C_13_H_21_NO](https://pubchem.ncbi.nlm.nih.gov/#query=C13H21NO) |  |
|  |  | gamma.-Tocopherol  (Phenolic compound derivative) | 36.798 | 0.313 | 416.365 | [C_28_H_48_O_2_](https://pubchem.ncbi.nlm.nih.gov/#query=C28H48O2) | 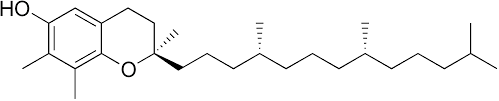 |
|  |  | Vitamin E  (Phenolic compound derivative) | 37.492 | 1.095 | 430.381 | [C_29_H_50_O_2_](https://pubchem.ncbi.nlm.nih.gov/#query=C29H50O2) | 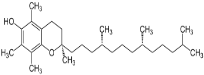 |
|  |  | 2-Ethylacridine  (Aromatic heterocyclic derivative) | 37.902 | 0.107 | 207.105 | [C_15_H_13_N](https://pubchem.ncbi.nlm.nih.gov/#query=C15H13N) |  |
|  |  | Propanamide, N-(4-methoxyphenyl)-2,2-dimethyl-  (Carboxylic acid derivative) | 38.438 | 0.371 | 207.126 | [C_12_H_17_NO_2_](https://pubchem.ncbi.nlm.nih.gov/#query=C12H17NO2) |  |
|  |  | 1,2,5-Oxadiazol-3-amine, 4-(4-methoxyphenoxy)-  (Amine derivative) | 38.59 | 0.257 | 207.064 | [C_9_H_9_N_3_O_3_](https://pubchem.ncbi.nlm.nih.gov/#query=C9H9N3O3) |  |
|  |  | Obtusifoliol  (Sterol derivative) | 38.945 | 1.309 | 426.386 | [C_30_H_50_O](https://pubchem.ncbi.nlm.nih.gov/#query=C30H50O) | 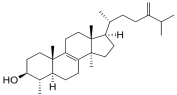 |
|  |  | beta-Sitosterol  (Sterol derivative) | 39.174 | 3.491 | 414.386 | [C_29_H_50_O](https://pubchem.ncbi.nlm.nih.gov/#query=C29H50O) | 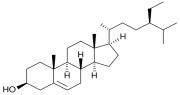 |
|  |  | Lanosterol  (Sterol derivative aka Phytosterol) | 39.48 | 6.595 | 426.386 | [C_30_H_50_O](https://pubchem.ncbi.nlm.nih.gov/#query=C30H50O) | 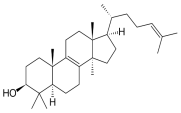 |
|  |  | 9,19-Cyclolanost-24-en-3-ol, (3.beta.)-  (Sterol derivative) | 39.82 | 24.963 | 426.386 | [C_30_H_50_O](https://pubchem.ncbi.nlm.nih.gov/#query=C30H50O) | 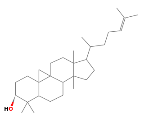 |
|  |  | 9,19-Cyclolanostan-3-ol, 24-methylene-, (3.beta.)-  (Sterol derivative aka Phytosterol) | 40.334 | 30.723 | 440.402 | [C_31_H_52_O](https://pubchem.ncbi.nlm.nih.gov/#query=C31H52O) | 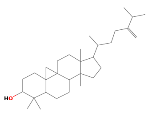 |
|  |  | 2,4-Dimethylbenzo[H]quinoline  (Alkaloid derivative) | 40.897 | 0.437 | 207.27 | [C_15_H_13_N](https://pubchem.ncbi.nlm.nih.gov/#query=C15H13N) |  |
|  |  | Benzoic acid, hexadecyl ester  (Ester derivative) | 41.731 | 1.667 | 346.287 | [C_23_H_38_O_2_](https://pubchem.ncbi.nlm.nih.gov/#query=C23H38O2) |  |

**Table 3:** The most important bioactive identified phytochemical compounds detected in the wild plant *Retama raetam*.

The plant was collected from the Tabuk Region between December 2022 and January 2023. Chemical screening of the entire aerial parts of the plant was performed utilizing GC-MS analysis. RT; retention time, MW; molecular weight, comp.; compound.

| **Plant Species** | **Total comp.** | **Most Bioactive Identified Components** | **Rt min** | **Score %** | **MW** | **Mol. Formula** | **Structure** |
| --- | --- | --- | --- | --- | --- | --- | --- |
| *Retama raetam* | 13 | Cathinone  (Monoamine alkaloid) | 1.234 | 1.96 | 149.084 | [C_9_H_11_NO](https://pubchem.ncbi.nlm.nih.gov/#query=C9H11NO) | 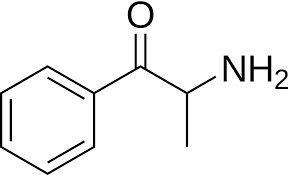 |
|  |  | Dodecahydro-7,14-methanodipyr  Ido[1,2-a:1',2'-e][1,5]diazocine  (Alkaloid derivative) | 22.414 | 77.311 | 234.21 | C_15_H_26_N_2_ | 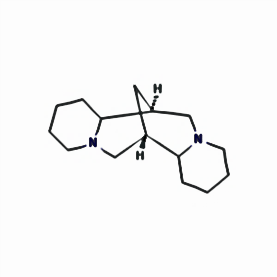 |
|  |  | 2-(2(3H)-Oxo-4H-1,4-benzoxazin-4-yl)acetic acid  (Acid derivative) | 25.444 | 1.949 | 207.053 | C_10_H_9_NO_4_ |  |
|  |  | Caulophylline  (Alkaloid derivative) | 26.118 | 2.43 | 204.126 | C_12_H_16_N_2_O | 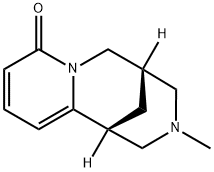 |
|  |  | 7,14-Methano-4H,6H-dipyrido  [1,2-a:1',2'-e][1,5]diazocin-4-one, 2,3,7,7a,8,9,10,11,13,14-decahy  dro-, [7S-(7.alpha.,7a.beta.,14.  alpha.)]-  (Alkaloid derivative) | 27.924 | 0.747 | 246.173 | C_15_H_22_N_2_O | 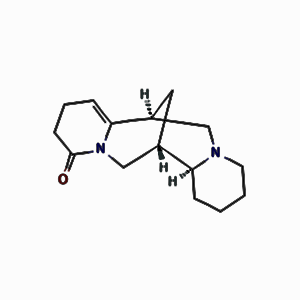 |
|  |  | Lupanine  (Alkaloid derivative) | 28.418 | 1.419 | 248.189 | C_15_H_24_N_2_O | 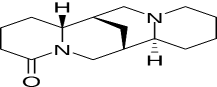 |
|  |  | 7,14-Methano-4H,6H-dipyrido  [1,2-a:1',2'-E][1,5]diazocin-4-one, 7,7a,8,9,10,11,13,14-octahydro-, [7R-(7.alpha.,7a.alpha.,14.alpha.)]-  (Alkaloid derivative) | 31.579 | 2.688 | 244.158 | C_15_H_20_N_2_O | 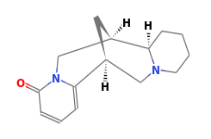 |
|  |  | Vitamin E  (Phenolic compound derivative) | 37.506 | 0.358 | 430.381 | C_29_H_50_O_2_ | 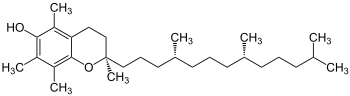 |
|  |  | Stigmasterol  (Sterol derivative) | 38.688 | 0.435 | 412.371 | C_29_H_48_O | 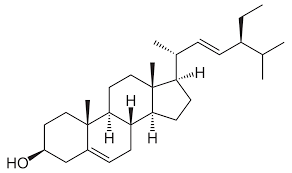 |
|  |  | beta.-Sitosterol  (Sterol derivative) | 39.181 | 0.524 | 414.386 | C_29_H_50_O | 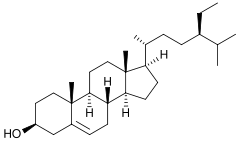 |

**Table 4:** The most important bioactive identified phytochemical compounds detected in *Artemisia monosperma*.

The plant was collected from the Tabuk Region between December 2022 and January 2023. Chemical screening of the entire aerial parts of the plant was performed utilizing GC-MS analysis. RT; retention time, MW; molecular weight, comp.; compound.

| **Plant Species** | **Total comp.** | **Most Bioactive Identified Components** | **Rt min** | **Score %** | **MW** | **Mol. Formula** | **Structure** |
| --- | --- | --- | --- | --- | --- | --- | --- |
| *Artemisia monosperma* | 32 | 2-Methylamino-N-phenyl-acetamide  (Carboxylic acid derivative) | 1.221 | 23.501 | 164.095 | [C_9_H_12_N_2_O](https://pubchem.ncbi.nlm.nih.gov/#query=C9H12N2O) |  |
|  |  | 3-Hydroxy-N-methylphenethylamine  (Phenolic compound) | 19.634 | 0.044 | 151.1 | [C_9_H_13_NO](https://pubchem.ncbi.nlm.nih.gov/#query=C9H13NO) |  |
|  |  | 3-(dimethylamino)-3-(1-piperidinyl)-2-Propenal  (Alkaloid derivative) | 20.899 | 2.707 | 182.142 | [C_10_H_18_N_2_O](https://pubchem.ncbi.nlm.nih.gov/#query=C10H18N2O) |  |
|  |  | 1-Methylpyrrolidine  (Alkaloid derivative) | 21.219 | 0.671 | 85.089 | [C_5_H_11_N](https://pubchem.ncbi.nlm.nih.gov/#query=C5H11N) |  |
|  |  | 4-ethynyl-1,3,5-trimethyl- Piperidin-4-ol  (Alkaloid derivative) | 21.316 | 0.449 | 167.131 | [C_10_H_17_NO](https://pubchem.ncbi.nlm.nih.gov/#query=C10H17NO) |  |
|  |  | 2,3,4-Trimethoxybenzoic acid  (Aromatic acid derivative) | 23.011 | 3.315 | 212.068 | [C_10_H_12_O_5_](https://pubchem.ncbi.nlm.nih.gov/#query=C10H12O5) | 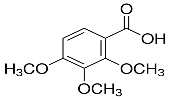 |
|  |  | 7a,9c-(Iminoethano)phenanthro[4,5-bcd]furan, 4a.alpha.,5-dihydro-3-methoxy-12-methyl-  (Alkaloid derivative) | 35.234 | 0.365 | 281.142 | [C_18_H_19_NO_2_](https://pubchem.ncbi.nlm.nih.gov/#query=C18H19NO2) | 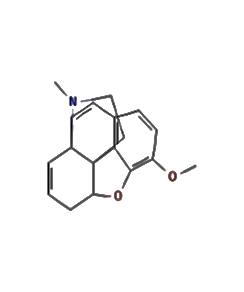 |
|  |  | Pyrido[2,3-d]pyrimidine, 4-phenyl-  (Alkaloid derivative) | 35.345 | 1.056 | 207.08 | [C_13_H_9_N_3_](https://pubchem.ncbi.nlm.nih.gov/#query=C13H9N3) |  |
|  |  | Hexahydropyridine, 1-methyl-4-[4,5-dihydroxyphenyl]-  (Alkaloid derivative) | 37.159 | 2.518 | 207.126 | [C_12_H_17_NO_2_](https://pubchem.ncbi.nlm.nih.gov/#query=C12H17NO2) |  |
|  |  | Vitamin E (Tocopherol)  (Phenolic compound derivative) | 37.513 | 4.404 | 430.381 | [C_29_H_50_O_2_](https://pubchem.ncbi.nlm.nih.gov/#query=C29H50O2) | 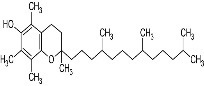 |
|  |  | 2-Ethylacridine  (Aromatic hydrocarbon derivative) | 38.75 | 0.538 | 207.105 | [C_15_H_13_N](https://pubchem.ncbi.nlm.nih.gov/#query=C15H13N) |  |
|  |  | gamma-Sitosterol  (Sterol derivative) | 39.174 | 18.611 | 414.386 | [C_29_H_50_O](https://pubchem.ncbi.nlm.nih.gov/#query=C29H50O) | 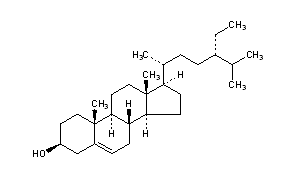 |
|  |  | Benzo[h]quinoline, 2,4-dimethyl-  (Quinoline derivative) | 41.905 | 3.356 | 207.105 | [C_15_H_13_N](https://pubchem.ncbi.nlm.nih.gov/#query=C15H13N) |  |

**Table 5:** The most important bioactive identified phytochemical compounds detected in the wild plant *Tamarix gallica*.

The plant was collected from the Tabuk Region between December 2022 and January 2023. Chemical screening of the entire aerial parts of the plant was performed utilizing GC-MS analysis. RT; retention time, MW; molecular weight, comp.; compound.

| **Plant Species** | **Total comp.** | **Most Bioactive Identified Components** | **Rt min** | **Score %** | **MW** | **Mol. Formula** | **Structure** |
| --- | --- | --- | --- | --- | --- | --- | --- |
| *Tamarix gallica* | 43 | Benzeneethanamine, 4-methoxy-.alpha.-methyl-  (Amine derivative) | 1.221 | 3.529 | 165.115 | C_10_H_15_NO |  |
|  |  | Bicyclo[2.2.1]heptane, 2,2-dimethyl-3-methylene-, (1R)-  (Hydrocarbon derivative) | 16.827 | 0.326 | 136.125 | C_10_H_16_ | 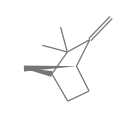 |
|  |  | 3-Buten-2-one, 3-methyl-4-(1,3,3-trimethyl-7-oxabicyclo[4.1.0]heptan-1-yl)-  (Ketone derivative) | 22.49 | 0.784 | 222.162 | C_14_H_22_O_2_ |  |
|  |  | Ethyl hydrogen malonate  (Ester derivative) | 24.061 | 0.588 | 132.042 | [C_5_H_8_O_4_](https://pubchem.ncbi.nlm.nih.gov/#query=C5H8O4) |  |
|  |  | 2-Thiophenecarboxylic acid, tetrahydro-3-hydroxy-3-methyl-5-phenyl-  (Ester derivative) | 26.277 | 7.216 | 238.066 | [C_12_H_14_O_3_S](https://pubchem.ncbi.nlm.nih.gov/#query=C12H14O3S) |  |
|  |  | Ledene alcohol  (Nonaromatic alcohol derivative) | 26.687 | 2.578 | 220.183 | C_15_H_24_O | 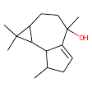 |
|  |  | p-Dimethylaminobenzylidene p-anisidine  (Aromatic amine derivative) | 29.126 | 13.214 | 254.142 | [C_16_H_18_N_2_O](https://pubchem.ncbi.nlm.nih.gov/#query=C16H18N2O) |  |
|  |  | 3-(3,4-dichlorophenyl)-1,1-dipropyl- Urea  (Amide derivative) | 30.572 | 5.075 | 288.08 | [C_13_H_18_Cl_2_N_2_O](https://pubchem.ncbi.nlm.nih.gov/#query=C13H18Cl2N2O) |  |
|  |  | Nithiamide  (Amide derivative) | 31.134 | 0.643 | 187.005 | [C_5_H_5_N_3_O_3_S](https://pubchem.ncbi.nlm.nih.gov/#query=C5H5N3O3S) |  |
|  |  | Androst-5-en-17-one, 3-(acetyloxy)-19-hydroxy-, (3.beta.)-  (Steroid derivative) | 31.663 | 15.009 | 346.214 | [C_21_H_30_O_4_](https://pubchem.ncbi.nlm.nih.gov/#query=C21H30O4) |  |
|  |  | Benzo[h]quinoline, 2,4-dimethyl-  (Alkaloid derivative) | 36.798 | 0.266 | 207.105 | [C_15_H_13_N](https://pubchem.ncbi.nlm.nih.gov/#query=C15H13N) |  |
|  |  | Vitamin E  (Phenolic compound derivative) | 37.506 | 0.377 | 430.381 | [C_29_H_50_O_2_](https://pubchem.ncbi.nlm.nih.gov/#query=C29H50O2) | 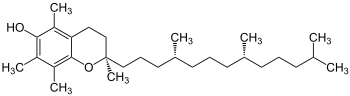 |
|  |  | Stigmasterol  (Phytosterol derivative) | 38.681 | 1.899 | 412.371 | [C_29_H_48_O](https://pubchem.ncbi.nlm.nih.gov/#query=C29H48O) | 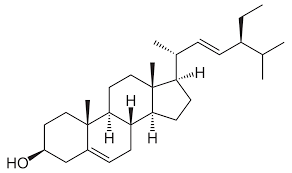 |
|  |  | beta.-Sitosterol  (Phytosterol derivative) | 39.181 | 2.246 | 414.386 | [C_29_H_50_O](https://pubchem.ncbi.nlm.nih.gov/#query=C29H50O) | 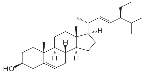 |
|  |  | 2-p-Nitrophenyl-5-isopropyloxy-oxadiazole-1,3,4  (Nitrobenzene derivative) | 39.577 | 0.207 | 249.075 | [C_11_H_11_N_3_O_4_](https://pubchem.ncbi.nlm.nih.gov/#query=C11H11N3O4) |  |
|  |  | 2-Ethylacridine  (Aromatic hydrocarbon derivative) | 40.661 | 1.156 | 207.105 | [C_15_H_13_N](https://pubchem.ncbi.nlm.nih.gov/#query=C15H13N) | 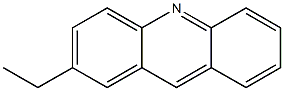 |
|  |  | 3-Methyl-sulfonylthiophene-2-carboxanilide  (Amide derivative) | 41.384 | 2.062 | 281.018 | [C_12_H_11_NO_3_S_2_](https://pubchem.ncbi.nlm.nih.gov/#query=C12H11NO3S2) |  |

**Table 6:** The most important bioactive identified phytochemical compounds detected in the wild plant *Artemisia judaica*.

The plant was collected from the Tabuk Region between December 2022 and January 2023. Chemical screening of the entire aerial parts of the plant was performed utilizing GC-MS analysis. RT; retention time, MW; molecular weight, comp.; compound.

| **Plant Species** | **Total comp.** | **Most Bioactive Identified Components** | **Rt min** | **Score %** | **MW** | **Mol. Formula** | **Structure** |
| --- | --- | --- | --- | --- | --- | --- | --- |
| *Artemisia judaica* | 28 | Acetic acid, hydroxy[(1-oxo-2-propenyl)amino]-  (Carboxylic derivative) | 1.22 | 1.974 | 145.038 | [C_5_H_7_NO_4_](https://pubchem.ncbi.nlm.nih.gov/#query=C5H7NO4) |  |
|  |  | Lilac alcohol D (Also known as 2-Furanethanol, 5-ethenyltetrahydro-β,5-dimethyl-, (βS,2R,5S)-;)  (Alcohol derivative) | 21.253 | 2.149 | 170.131 | [C_10_H_18_O_2_](https://pubchem.ncbi.nlm.nih.gov/#query=C10H18O2) | 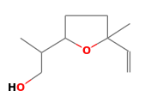 |
|  |  | 4,7-Methanofuro[3,2-c]oxacycloundecin-6(4H)-one, 7,8,9,12-tetrahydro-3,11-dimethyl-  (Ketone/lactone/furan derivative) | 26.006 | 2.076 | 246.126 | [C_15_H_18_O_3_](https://pubchem.ncbi.nlm.nih.gov/#query=C15H18O3) | 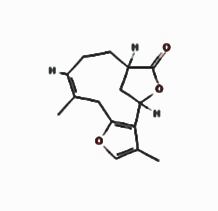 |
|  |  | Alantolactone, 4.alpha.,4A.alpha.-epoxy-  (Lactone derivative) | 26.472 | 0.824 | 248.141 | [C_15_H_20_O_3_](https://pubchem.ncbi.nlm.nih.gov/#query=C15H20O3) |  |
|  |  | Naphtho[1,2-b]furan-2,6(3H,4H)-dione, 3a,5,5a,9,9a,9b-hexahydro-9-hydroxy-3,5a,9-trimethyl-  (Ketone/lactone derivative) | 27.34 | 51.299 | 264.136 | [C_15_H_20_O_4_](https://pubchem.ncbi.nlm.nih.gov/#query=C15H20O4) |  |
|  |  | Benzoic acid, 3,5-bis(1,1-dimethylethyl)-4-hydroxy-, methyl ester  (Phenol derivative) | 27.431 | 14.227 | 264.173 | [C_16_H_24_O_3_](https://pubchem.ncbi.nlm.nih.gov/#query=C16H24O3) | 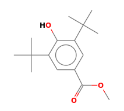 |
|  |  | 2(1H)-Naphthalenone, 3,4,4a,5,6,7,8,8a.alpha.-octahydro-5.alpha.-hydroxy-4a.alpha.,7,7-trimethyl-, acetate  (Ketone derivative) | 29.293 | 0.810 | 252.173 | [C_15_H_24_O_3_](https://pubchem.ncbi.nlm.nih.gov/#query=C15H24O3) |  |
|  |  | Propanedioic acid, (phenylmethyl)-, diethyl ester (Also known as Diethyl benzylmalonate)  (Ester/benzene derivative) | 30.328 | 0.397 | 250.121 | [C_14_H_18_O_4_](https://pubchem.ncbi.nlm.nih.gov/#query=C14H18O4) | 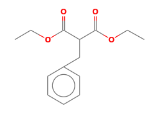 |
|  |  | 2-Propenoic acid, 3-phenyl-, ethyl ester, (E)-  (Ester derivative) | 30.662 | 0.219 | 176.084 | [C_11_H_12_O_2_](https://pubchem.ncbi.nlm.nih.gov/#query=C11H12O2) | 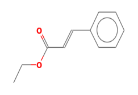 |
|  |  | Vitamin E  (Phenol derivative) | 37.492 | 0.091 | 430.381 | [C_29_H_50_O_2_](https://pubchem.ncbi.nlm.nih.gov/#query=C29H50O2) | 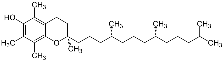 |
|  |  | Stigmasterol  (Sterol derivative) | 38.666 | 0.682 | 412.371 | [C_29_H_48_O](https://pubchem.ncbi.nlm.nih.gov/#query=C29H48O) | 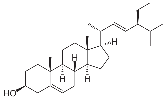 |
|  |  | .gamma.-Sitosterol  (Sterol derivative) | 414.386 | 39.167 | 0.678 | [C_29_H_52_O](https://pubchem.ncbi.nlm.nih.gov/#query=C29H52O2) | 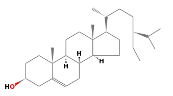 |
|  |  | Pyridine-3-carboxamide, oxime, N-(2-trifluoromethylphenyl)-  (Oxime derivative) | 40.619 | 0.491 | 281.078 | [C_13_H_10_F_3_N_3_O](https://pubchem.ncbi.nlm.nih.gov/#query=C13H10F3N3O) |  |
